# Spatial Climate Patterns Explain Negligible Variation in Strength of Compensatory Density Feedbacks in Birds and Mammals

**DOI:** 10.1371/journal.pone.0091536

**Published:** 2014-03-11

**Authors:** Salvador Herrando-Pérez, Steven Delean, Barry W. Brook, Phillip Cassey, Corey J. A. Bradshaw

**Affiliations:** 1 The Environment Institute and School of Earth and Environmental Sciences, University of Adelaide, South Australia, Australia; 2 Department of Biogeography and Global Change, National Museum of Natural Sciences, Spanish Research Council (CSIC), Madrid, Spain; Columbia University, United States of America

## Abstract

The use of long-term population data to separate the demographic role of climate from density-modified demographic processes has become a major topic of ecological investigation over the last two decades. Although the ecological and evolutionary mechanisms that determine the strength of density feedbacks are now well understood, the degree to which climate gradients shape those processes across taxa and broad spatial scales remains unclear. Intuitively, harsh or highly variable environmental conditions should weaken compensatory density feedbacks because populations are hypothetically unable to achieve or maintain densities at which social and trophic interactions (e.g., competition, parasitism, predation, disease) might systematically reduce population growth. Here we investigate variation in the strength of compensatory density feedback, from long-term time series of abundance over 146 species of birds and mammals, in response to spatial gradients of broad-scale temperature precipitation variables covering 97 localities in 28 countries. We use information-theoretic metrics to rank phylogenetic generalized least-squares regression models that control for sample size (time-series length) and phylogenetic non-independence. Climatic factors explained < 1% of the remaining variation in density-feedback strength across species, with the highest non-control, model-averaged effect sizes related to extreme precipitation variables. We could not link our results directly to other published studies, because ecologists use contrasting responses, predictors and statistical approaches to correlate density feedback and climate – at the expense of comparability in a macroecological context. Censuses of multiple populations within a given species, and *a priori* knowledge of the spatial scales at which density feedbacks interact with climate, seem to be necessary to determine cross-taxa variation in this phenomenon. Despite the availability of robust modelling tools, the appropriate data have not yet been gathered for most species, meaning that we cannot yet make any robust generalisations about how demographic feedbacks interact with climate.

## Introduction

*The quality of the food or the temperature prevailing, however, may have an important effect upon the level at which a population is adjusted by governing factors [1]*

The interplay of density-independent (environmental forcing via weather, climate, or food supply) and density-dependent (i.e., trophic and social interactions between individuals) drivers of population change through long-term time series of population abundance has become a major area of ecological research over the last two decades [2], [3]. Many of those studies concentrate on single species (e.g., [4]–[6]), while cross-taxa patterns have only been compared for a few phylogenetically and/or trophically related species, with increasing attention being devoted to the strength of density feedbacks using a variety of modelling methods (e.g., [7]–[13]). After much research effort and impressive mathematical development to deal with global patterns of population dynamics (e.g., [13]), we still lack an understanding of how the demographic role of density feedbacks varies among species over broad climate gradients. Ecologists can even question whether those gradients are biologically meaningful, and hence detectable.

For relatively well-studied taxa such as large herbivores, there is a general appreciation that the strength of ‘compensatory density feedback’ [14] should be milder in harsher environments [15] because harsh climate conditions should prevent populations from reaching densities at which social and trophic interactions might strongly reduce population growth. Theoretical postulates are disparate about supporting the former prediction. Thus, because the metabolic rates of organisms are shaped by environmental temperature, metabolic theory proposes that the rates of resource exploitation, and therefore the intensity of mechanisms modified by compensatory density feedbacks (e.g., competition, disease, parasitism, predation,), should be higher in warmer (i.e., more benign) environments [16]. In contrast, Wilmers et al. [17] have modelled that the accumulation of years of benign environmental conditions can gradually promote fertility, recruitment and survival, but populations are then more vulnerable to compensatory density feedbacks when harsh environmental conditions return. Those density feedbacks are likely to occur because long-term climate patterns alter carrying capacities and the magnitude of competition for vital resources such as food or water; yet the analyses of time series of abundance incorporating shifts in climate variables as proxies for carrying capacity have only been applied to few species (e.g., [6], [18]). In the only cross-taxa study correlating density-feedback strength (from time series of abundance) explicitly with broad climate variables (single, average values per population), Wang et al. [19] showed that for two large ungulates (bison *Bison bison* and elk *Cervus elaphus*) in North America, colder winter temperature and temporal heterogeneity accentuated the model-averaged compensatory density-feedback strength, i.e., feedbacks were in fact stronger in response to harsher (i.e., colder or more variable) climates.

Herein, we expand the taxonomic coverage of the analytical approach of Wang et al. [19] over 146 bird and mammal species comprising slow to fast life histories and a spatial extent of 97 localities in 28 countries. We hypothesize that broad climate variables can explain variation in compensatory density feedbacks across species. We quantified such variation by collating a dataset comprising geo-referenced, long-term censuses of population size and average temperature and precipitation in the last five decades at a coarse spatial resolution (21 km^2^). We also revise caveats in quantifying global patterns of density feedback.

## Materials and Methods

### Data

We used year-round time series of abundance of 91 birds and 55 mammal species (1 population per species) from two previous studies [20], [21]). Of those studies, we had retained time series of well-defined populations, mostly resident in their native ranges or, for migratory birds, in their breeding localities; we excluded populations from the smallest oceanic islands (e.g., Gough, Cosin, Marion) and remote areas (e.g., Antarctica) for which we could not obtain adequate climatic data (see below). Our dataset had a median time-series length of 26 years with 95^th^ percentile range of 10 to 97 years, and represented 97 different localities in 28 countries (see map in Figure S1 in File S1, and time series of abundance in File S2) – 88% of the populations were from the Northern Hemisphere (mainly Europe and North America). The species covered body lengths (from beak/nose tip to tail end) between 90 and 1520 mm in birds, and between 24 and 5000 mm in mammals – the distributions of (log-transformed) body lengths was nearly symmetric, indicating no bias towards neither end of the body-size spectrum.

We geo-referenced the locality of each population as the latitude/longitude reported in the ecological literature from which we retrieved the census data. For each population’s geographic position, we collated one broad estimate of four environmental variables from the *Bioclim* suite (www.worldclim.org), which represented annual trends and seasonality between 1950 and 2000: (*i*) mean annual temperature (°C), (*ii*) temperature seasonality (sd, °C), (*iii*) mean annual precipitation (mm), and (*iv*) precipitation seasonality (CV, mm). These estimates are derived from monthly data collected by weather stations at a 1-km^2^ resolution. They are calculated as follows: (*i*) temperature (or precipitation) estimates at month *i* are averaged across meteorological stations within a given spatial resolution and (*ii*) annual estimates at year *i* are the mean of monthly estimates. Thus, our model variables are means, standard deviations (or coefficients of variation) of annual estimates of temperature (or precipitation) for 1950–2000 [22]. These data are widely used in species distribution models (e.g., [23], [24]). We used interpolated data with 2.5° resolution (21 km^2^) [22] because that was the prevailing resolution of our population data. The magnitudes of each environmental variable at 2.5 ° resolution and 5 ° (42 km^2^) or 10 ° (84 km^2^) resolutions were strongly correlated (all Spearman *ρ*>0.99), so we are confident that our results are consistent at those three spatial scales. To assess the representation of global climates in our dataset, we categorized our localities according to the Köppen-Geiger classification [25] by entering their latitude and longitude in a climate layer superimposed in *Google Earth* [26] – see Results and Supporting Dataset (File S2).

To satisfy the assumptions of time-series analyses, and minimize the confounding effects of measurement error, authors *either* set stringent criteria for data selection [13], [27], *or* use state-space models [28]–[30], which themselves are not, however, exempt of caveats [30] and add further model complexity to cross-taxa comparisons. We replicated all analyses for the total sample of 146 species (as above), and a subset of 120 species (76 birds, 44 mammals) from 94 localities and 26 countries, after meeting stringent criteria of stationarity, trending, outliers and missing values following Herrando-Pérez et al. [20]. These criteria are explained in Supporting Information (File S1). For the core dataset from which most of our series was extracted, Herrando-Pérez et al. [20] showed the same model rankings and similar goodness of fit in observed and simulated time series after incorporating 5% measurement error in birds and 10% in mammals.

### Density feedback

We estimated strength of density feedback through the Gompertz model [31], [32], i.e., the slope of the relationship of the intrinsic growth rate *r*  =  log*_e_*(*N_t_*_+1_/*N_t_*)) versus population size on a log scale, such that *r*  =  *α* + *β* log_e_ (*N_t_*) + *ε_t_*, where *r*  =  proportional change in population size between consecutive time steps, *N_t_*  =  the population size at time *t, α*  =  intercept, *β*  =  strength of density feedback on *r*, and *ε_t_*  =  Gaussian random variable with a mean of zero and a variance *σ* ^2^ reflecting uncorrelated stochastic variability in *r*. Feedback strength in this model expresses change in *r* between *t* and *t*+1 per unit change in log population size (i.e., through the interplay of component density feedbacks on survival and fertility rates [33]).

There are four important aspects of our study to consider: (1) we focused only on compensatory density feedbacks occurring between consecutive years, which are common signals of intra-specific competition for food resources [34], and so we did not investigate delayed feedbacks occurring every other year (or longer time lags) that are often attributed to predators and parasites [34], [35]; (2) the Gompertz model is a measure of compensation on a proportional (logarithmic) scale, thus it is invariant to the absolute value of the carrying capacity of the environment, and no scaling is required to compare density-feedback strength across species; (3) we measured density feedbacks, but did not test for population regulation. Density feedback and population regulation are not synonymous [14]; therefore, our results are not comparable to studies examining regulatory dynamics. Such studies often show contradictory results even from the same datasets because authors use different models to produce rival conceptualizations of regulation (e.g., [36], [37]); (4) finally, Knape and de Valpine [13] used a linear autoregressive model incorporating time series of *both* population abundance *and* climate, whereas we only used time series of abundance and one single, average estimate of each of the four climate variables at each study site. In so doing, we are testing a different hypothesis from that examined by Knape and de Valpine [13] (see below and Discussion).

Because the Gompertz model is measured on a proportional scale, it characterizes the multiplicative nature of demographic rates better than the Ricker-logistic model (linear relationship of *r* to density) [38], and unequivocally informs the magnitude of the compensatory response of demographic rates to changes in population size relative to nonlinear models [39]. Furthermore, the Gompertz model has performed robustly in describing the general dynamics of populations of birds and mammals over a wide range of body sizes (e.g., [40]–[47]), is present in multi-model inference scenarios where competing models are contrasted [6], [18], [42], [48], [49], is the top-ranked model in meta-analyses of hundreds of species in which various alternatives have also been evaluated (e.g., [50]), and has been used in theoretical development about density feedback (e.g., [28]). We avoided fitting the fully parameterized *θ*-logistic model [i.e., Ricker-logistic model with a shape parameter allowing for a non-linear tendency to carrying capacity] due to recent caveats of application to analyses of time series [21], [51], or other highly parameterized analogues (e.g., hyperbolic growth). Most species were from temperate and polar regions, the demographic rates of which are subject to strong annual seasonality, so we deemed year-round demographic estimates appropriate measures of population turnover.

We stress that our study attempted to capture gross environmental gradients, not aspects of (regional) climate change. Further, although some time series of abundance extended a few years beyond the temporal window of the climate data (1950–2000), the overall average variation in temperature (or precipitation) carries regional variation across all regions notwithstanding. Moreover, there is no obvious way one could ‘standardize’ the climate metrics with sufficient justification to account for regional warming trends.

### Modelling

We included nine models in our model set (Table 1). With strength of compensatory density feedback (none of the target time series was depensating, so strengths were invariably negative) as the common response, the null model equated time-series length (control variable, see below), and the remaining eight models included a single environmental variable (four models), and each temperature variable with one of the two precipitation variables (four models). Temperature variables were poorly correlated with precipitation variables (Spearman *ρ* < |0.3| for all cross-paired correlations, Figure S2 in File S1). We converted the strengths of compensatory density feedback to the square root of their absolute (otherwise negative) values to meet model assumptions, which we checked in QQ and residual plots. Time-series length correlates positively with increasing statistical support for density feedback in multiple-species studies [50], so we accounted for this correlation by including this in all models. We log-transformed time-series length and the two precipitation variables to approximate a linear relationship with the response. We could not fit more complex models due to available sample size [52], while interaction terms between continuous variables would be of difficult to interpret in the context of our analyses (broad climate estimates across 28 countries). To account for extreme climatic conditions, we applied our approach to two additional model sets including another four *Bioclim* variables. We substituted mean temperature and precipitation by minimum temperature in the coldest month and precipitation in the driest month in the second set (Table 2), and by maximum temperature in the warmest month and precipitation in the wettest month in the third set (Table 3). We opted for three model sets, instead of one set with numerous variables, to minimize collinearity issues (Figure S2 in File S1). We present the raw values of the response and all variables in the Supporting Dataset (File S2).

**Table 1:** Density feedback and mean climate variables.

Taxa	Top-ranked models	*w*AIC*_c_*	%Variance	*ER*	Top rank
**birds**	Strength ∼ *q*	**0.29** [0.25, 0.31]	**3.6** [3.2, 4.2]	-	**100** (0)
	Strength ∼ *q* + mT	**0.13** [0.10, 0.16]	**3.6** [3.2, 4.2]	2.2	**0** (33)
	Strength ∼ *q* + mP	**0.13** [0.11, 0.15]	**3.6** [3.2, 4.2]	2.2	**0** (42)
	Strength ∼ *q* + sT	**0.12** [0.10, 0.16]	**3.6** [3.2, 4.2]	2.3	**0** (25)
	Strength ∼ *q* + sP	**0.10** [0.09, 0.10]	**3.6** [3.2, 4.2]	3.0	**0** (0)
	Strength ∼ *q* + mT + mP	**0.06** [0.04, 0.08]	**3.5** [3.1, 4.1]	5.0	**0** (0)
	Strength ∼ *q* + mT + sP	**0.05** [0.04, 0.06]	**3.6** [3.2, 4.2]	6.3	**0** (0)
	Strength ∼ *q* + sT + mP	**0.09** [0.07, 0.13]	**3.5** [3.1, 4.1]	3.3	**0** (0)
	Strength ∼ *q* + sT + sP	**0.04** [0.03, 0.05]	**3.6** [3.2, 4.2]	7.2	**0** (0)
**mammals**	Strength ∼ *q*	**0.12** [0.07, 0.20]	**2.1** [1.8, 2.5]	1.9	**4** (19)
	Strength ∼ *q* + mT	**0.24** [0.15, 0.37]	**1.8** [1.6, 2.3]	-	**82** (11)
	Strength ∼ *q* + mP	**0.04** [0.02, 0.06]	**2.1** [1.8, 2.5]	6.2	**0** (0)
	Strength ∼ *q* + sT	**0.11** [0.05, 0.21]	**1.9** [1.7, 2.3]	2.1	**3** (20)
	Strength ∼ *q* + sP	**0.11** [0.05, 0.22]	**1.9** [1.7, 2.3]	2.2	**7** (6)
	Strength ∼ *q* + mT + mP	**0.07** [0.05, 0.11]	**1.8** [1.6, 2.3]	3.5	**0** (0)
	Strength ∼ *q* + mT + sP	**0.13** [0.07, 0.22]	**1.8** [1.6, 2.2]	2.0	**2** (34)
	Strength ∼ *q* + sT + mP	**0.04** [0.02, 0.06]	**1.9** [1.7, 2.3]	6.5	**0** (0)
	Strength ∼ *q* + sT + sP	**0.12** [0.07, 0.20]	**1.8** [1.6, 2.1]	2.0	**2** (10)

**Figure 1:**
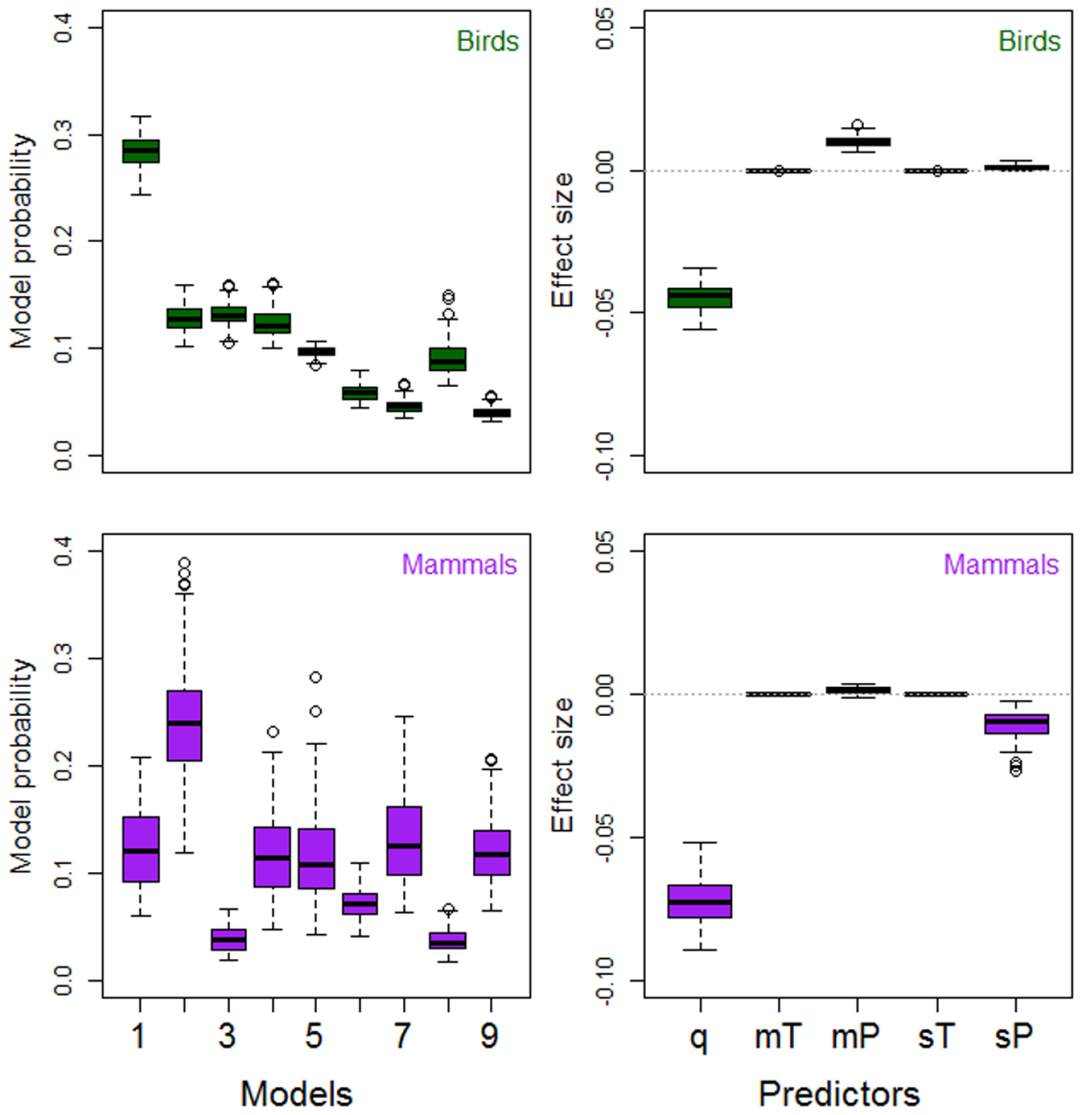
Density feedback and mean climate variables. Model probabilities (left panels; Table 1) and standardized *w*AIC*_c_*-averaged effect sizes (right panels; Table 4) result from contrasting 9 models with strength of compensatory density feedback from time series of abundance (response) and combinations of 6 explanatory variables including time-series length (*q*, years), mean annual temperature (mT, °C), mean annual precipitation (mP, mm), seasonality of temperature (sT  =  sd, °C) and seasonality of precipitation (sP  =  CV). Models were fitted as phylogenetic generalized least-squares regression for two datasets comprising 91 bird and 55 mammal species, respectively.

Akaike’s information criterion (AIC*_c_*) support for the model set correlating temperature and precipitation variables^1^ to strength of compensatory density feedback for birds (91 species) or mammals (55 species) (Figure 1). All models were fitted through phylogenetic generalized least-squares regression, and model-ranking descriptors (*w*AIC*_c_*, % variance and *ER*)^2^ are medians from 100 bootstrapped samples [95^th^ percentile ranges].

1 **mT**  =  mean annual temperature (°C), **mP  = ** mean annual precipitation (mm); **sT  = ** seasonality of temperature (sd, °C), and **sP  = ** seasonality of precipitation (CV). The model set equated *q* as control variable (i.e., present in all models), eight combinations of climate variables [mT | mP | sT | sP | mT+mP | mT+sP | sT+mP | sT+sP], and a null model with only *q* (time-series length, years).

2 ***w*** **AIC** ***_c_***  =  model probabilities given each dataset and model set; %**Variance**  =  % variance in density-feedback strength explained by each model within the set; ***ER***  =  evidence ratio of first model over the remaining models according to *w*AIC*_c_*; and **Top rank**  =  times each model was top-ranked over the 100 bootstrapped samples (times each model was second-ranked).

**Table 2:** Density feedback and minimum climate variables.

Taxa	Top-ranked models	*w*AIC*_c_*	%Variance	*ER*	Top rank
**birds**	Strength ∼ *q*	**0.24** [0.21, 0.27]	**3.6** [3.1, 4.2]	-	**78** (21)
	Strength ∼ *q* + minT	**0.13** [0.11, 0.15]	**3.6** [3.0, 4.1]	1.9	**0** (1)
	Strength ∼ *q* + minP	**0.21** [0.17, 0.25]	**3.5** [3.0, 4.1]	1.1	**20** (78)
	Strength ∼ *q* + sT	**0.10** [0.08, 0.13]	**3.6** [3.1, 4.2]	2.4	**3** (47)
	Strength ∼ *q* + sP	**0.08** [0.07, 0.09]	**3.6** [3.1, 4.2]	3.0	**0** (0)
	Strength ∼ *q* + minT + minP	**0.09** [0.07, 0.11]	**3.5** [3.0, 4.0]	2.6	**0** (0)
	Strength ∼ *q* + minT + sP	**0.04** [0.04, 0.06]	**3.6** [3.0, 4.1]	5.6	**0** (0)
	Strength ∼ *q* + sT + minP	**0.08** [0.07, 0.10]	**3.5** [3.0, 4.0]	2.8	**0** (0)
	Strength ∼ *q* + sT + sP	**0.03** [0.03, 0.04]	**3.6** [3.1, 4.2]	7.5	**2** (0)
**mammals**	Strength ∼ *q*	**0.16** [0.09, 0.23]	**2.1** [1.8, 2.4]	-	**24** (26)
	Strength ∼ *q* + minT	**0.13** [0.07, 0.19]	**1.9** [1.7, 2.2]	1.2	**5** (9)
	Strength ∼ *q* + minP	**0.06** [0.04, 0.08]	**2.0** [1.8, 2.4]	2.6	**0** (0)
	Strength ∼ *q* + sT	**0.15** [0.08, 0.24]	**1.9** [1.7, 2.2]	1.0	**28** (26)
	Strength ∼ *q* + sP	**0.13** [0.07, 0.26]	**1.9** [1.7, 2.3]	1.2	**19** (16)
	Strength ∼ *q* + minT + minP	**0.04** [0.03, 0.06]	**1.9** [1.6, 2.2]	3.5	**0** (0)
	Strength ∼ *q* + minT + sP	**0.11** [0.07, 0.18]	**1.8** [1.6, 2.0]	1.5	**1** (6)
	Strength ∼ *q* + sT + minP	**0.05** [0.03, 0.08]	**1.9** [1.7, 2.2]	2.9	**0** (0)
	Strength ∼ *q* + sT + sP	**0.14** [0.10, 0.22]	**1.8** [1.6, 2.0]	1.1	**23** (17)

**Figure 2:**
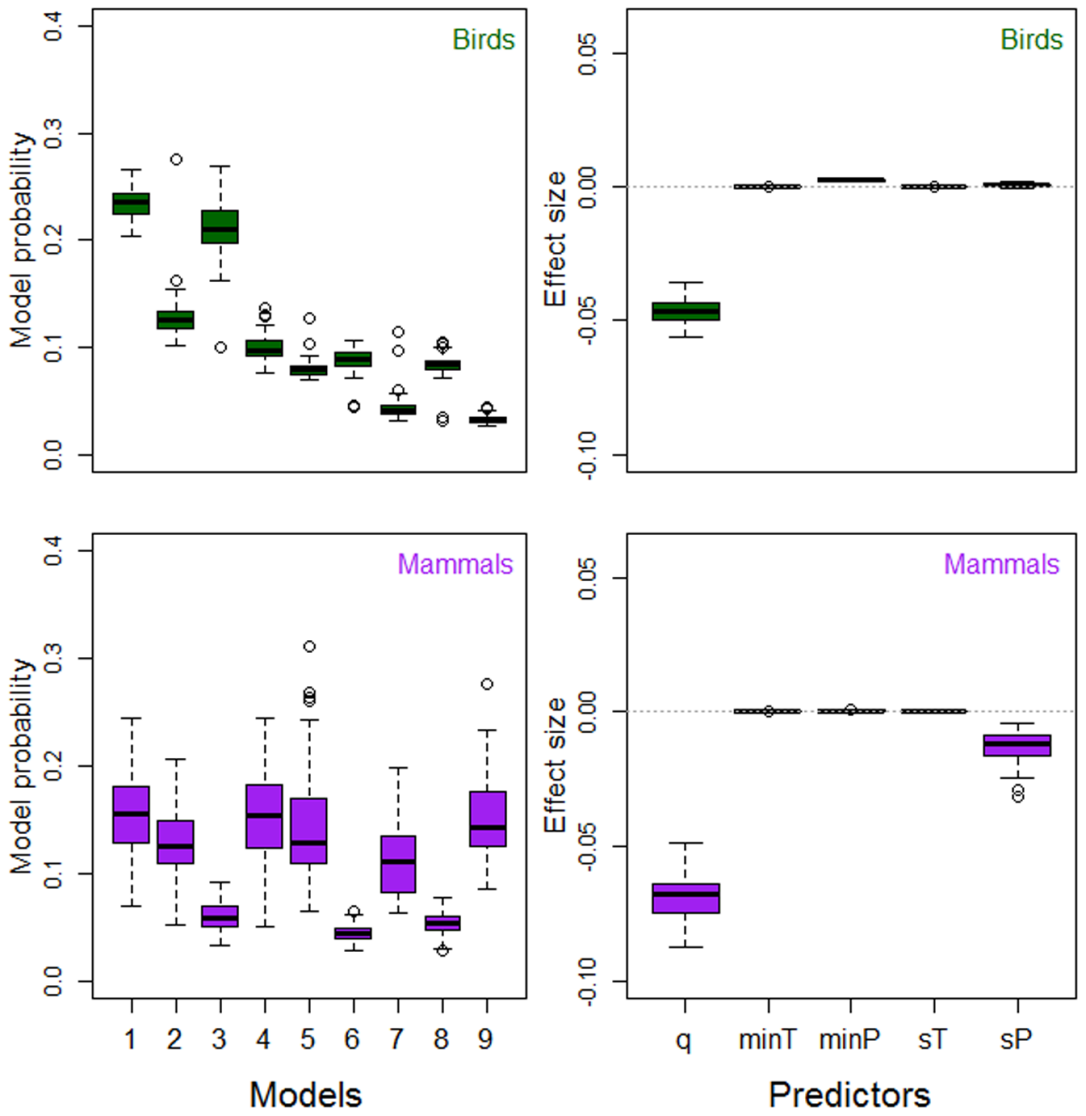
Density feedback and minimum climate variables. Model probabilities (left panels; Table 2) and standardized *w*AIC*_c_*-averaged effect sizes (right panels; Table 5) result from contrasting 9 models with strength of compensatory density feedback from time series of abundance (response) and combinations of 6 explanatory variables including time-series length (*q*, years), temperature of the coldest month (minT, °C), precipitation of the driest month (minP, mm), seasonality of temperature (sT  =  sd, °C) and seasonality of precipitation (sP  =  CV). Models were fitted as phylogenetic generalized least-squares regression for two datasets comprising 91 bird and 55 mammal species, respectively.

Akaike’s information criterion (AIC*_c_*) support for the model set correlating temperature and precipitation variables^1^ to strength of compensatory density feedback for birds (91 species) and mammals (55 species) (Figure 2). All models were fitted through phylogenetic generalized least-squares regression, and model-ranking descriptors (*w*AIC*_c_*, % variance and *ER*)^2^ are medians from 100 bootstrapped samples [95^th^ percentile ranges].

1 **minT**  =  temperature of the coldest month (°C), **minP  = ** precipitation of the driest month (mm); **sT  = ** seasonality of temperature (sd, °C), and **sP  = ** seasonality of precipitation (CV). The model set equated *q* as control variable (i.e., present in all models), eight combinations of climate variables [minT | minP | sT | sP | minT+minP | minT+sP | sT+minP | sT+sP], and a null model with only *q*.

2 Abbreviations of AIC*_c_* metrics are as in Table 1.

**Table 3:** Density feedback and maximum climate variables.

Taxa	Top-ranked models	*w*AIC*_c_*	%Variance	*ER*	Top rank
**birds**	Strength ∼ *q*	**0.24** [0.17, 0.28]	**3.7** [3.1, 4.3]	-	**69** (25)
	Strength ∼ *q* + maxT	**0.09** [0.06, 0.12]	**3.7** [3.1, 4.3]	2.6	**0** (0)
	Strength ∼ *q* + maxP	**0.17** [0.14, 0.20]	**3.7** [3.0, 4.2]	1.4	**2** (51)
	Strength ∼ *q* + sT	**0.10** [0.08, 0.12]	**3.6** [3.0, 4.2]	2.4	**0** (0)
	Strength ∼ *q* + sP	**0.08** [0.06, 0.09]	**3.7** [3.1, 4.3]	3.0	**0** (0)
	Strength ∼ *q* + maxT + maxP	**0.06** [0.04, 0.08]	**3.7** [3.0, 4.2]	4.0	**0** (0)
	Strength ∼ *q* + maxT + sP	**0.03** [0.02, 0.04]	**3.7** [3.0, 4.3]	7.2	**0** (0)
	Strength ∼ *q* + sT + maxP	**0.18** [0.11, 0.36]	**3.4** [2.9, 4.0]	1.4	**29** (2)
	Strength ∼ *q* + sT + sP	**0.03** [0.03, 0.04]	**3.6** [3.0, 4.2]	7.4	**0** (0)
**mammals**	Strength ∼ *q*	**0.13** [0.07, 0.19]	**2.1** [1.8, 2.4]	1.0	**12** (13)
	Strength ∼ *q* + maxT	**0.13** [0.08, 0.20]	**2.0** [1.7, 2.3]	-	**25** (14)
	Strength ∼ *q* + maxP	**0.12** [0.08, 0.16]	**2.0** [1.7, 2.3]	1.1	**1** (11)
	Strength ∼ *q* + sT	**0.13** [0.06, 0.23]	**2.0** [1.6, 2.3]	1.1	**12** (29)
	Strength ∼ *q* + sP	**0.11** [0.06, 0.21]	**2.0** [1.7, 2.2]	1.2	**18** (5)
	Strength ∼ *q* + maxT + maxP	**0.04** [0.02, 0.07]	**2.0** [1.7, 2.3]	3.4	**0** (0)
	Strength ∼ *q* + maxT + sP	**0.06** [0.03, 0.12]	**1.9** [1.6, 2.2]	2.4	**0** (0)
	Strength ∼ *q* + sT + maxP	**0.13** [0.07, 0.24]	**1.8** [1.5, 2.1]	1.1	**17** (15)
	Strength ∼ *q* + sT + sP	**0.12** [0.06, 0.22]	**1.8** [1.5, 2.0]	1.0	**15** (13)

**Figure 3:**
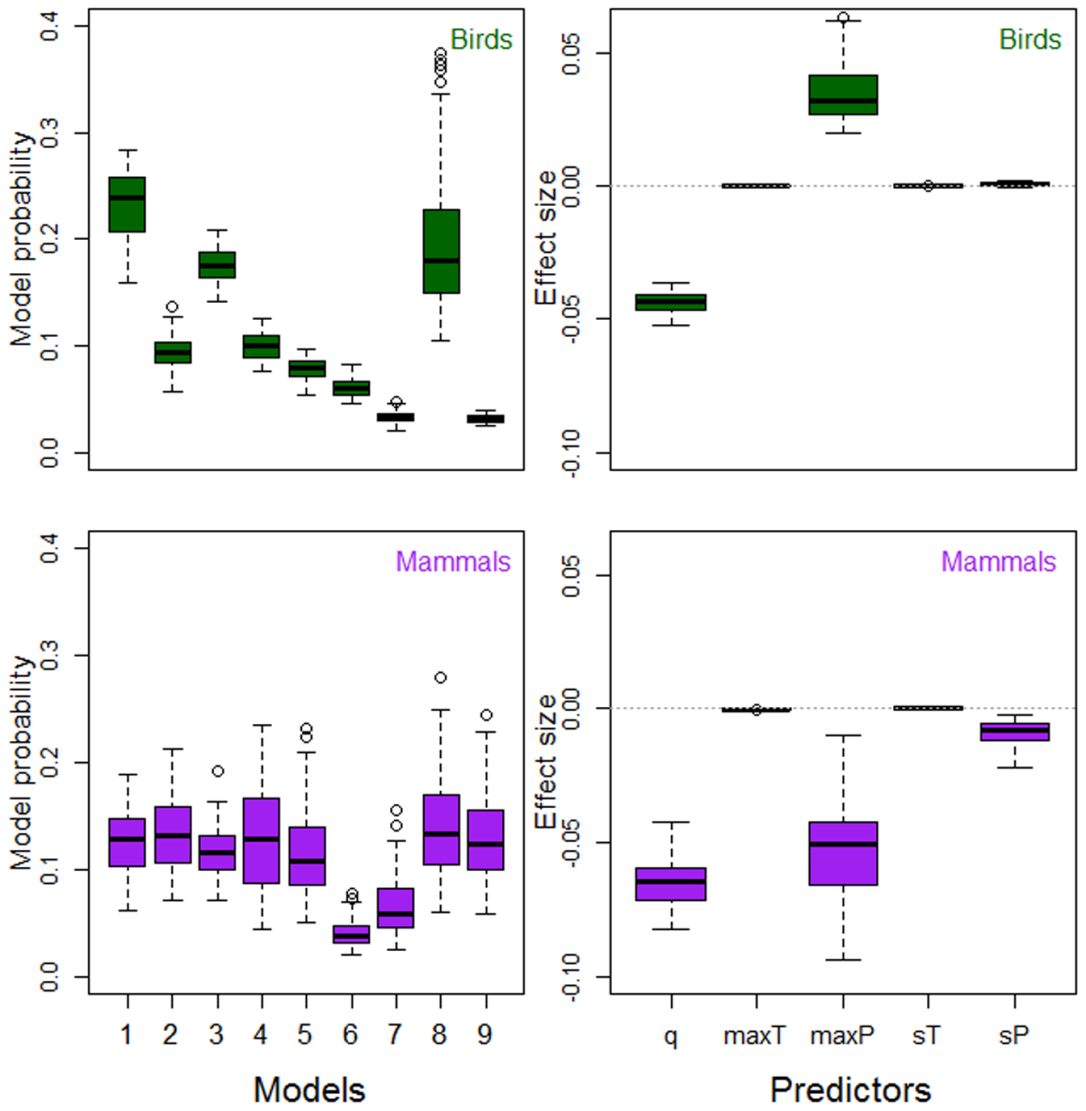
Density feedback and maximum climate variables. Model probabilities (left panels; Table 3) and standardized *w*AIC*_c_*-averaged effect sizes (right panels; Table 6) result from contrasting 9 models with strength of compensatory density feedback from time series of abundance (response) and combinations of 6 explanatory variables including time-series length (*q*, years), temperature of the hottest month (maxT, °C), precipitation of the wettest month (maxP, mm), seasonality of temperature (sT  =  sd, °C) and seasonality of precipitation (sP  =  CV). Models were fitted as phylogenetic generalized least-squares regression for two datasets comprising 91 bird and 55 mammal species, respectively.

Akaike’s information criterion (AIC*_c_*) support for the model set correlating temperature and precipitation variables^1^ to strength of compensatory density feedback for birds (91 species) or mammals (55 species) (Figure 3). All models were fitted through phylogenetic generalized least-squares regression, and model-ranking descriptors (*w*AIC*_c_*, % variance and *ER*)^2^ are medians from 100 bootstrapped samples [95^th^ percentile ranges].

1 **maxT**  =  temperature of the hottest month (°C), **maxP  = ** precipitation of the wettest month (mm); **sT  = ** seasonality of temperature (sd, °C), and **sP  = ** seasonality of precipitation (CV). The model set equated *q* as control variable (i.e., present in all models), eight combinations of climate variables [maxT | maxP | sT | sP | maxT+maxP | maxT+sP | sT+maxP | sT+sP], and a null model with only *q*.

2 Abbreviations of AIC*_c_* metrics are as in Table 1.

We fitted our data using phylogenetic generalized least-squares regression (PGLS). PGLS incorporates the phylogenetic covariance between taxa into the calculation of the effect sizes of the explanatory variables, and so accounts for the phylogenetic non-independence of species relatives [53], [54]. Phylogenetic relationships were based on recent species-level molecular phylogenies for birds [55] and mammals [56] pruned to the species available in our dataset. We ranked model support by means of Akaike’s information criteria corrected for finite sample size, AIC*_c_* [57]. We calculated model ranking and effect sizes on 100 bootstrapped samples of the response and explanatory variables measured in all species and localities (e.g., as in [20]). Bootstrapping accounted for the fact that, of the 97 study localities (see Methods, Figure S1 in File S1), each of 19 localities contributed time series of abundance from two to 12 species (47% of the dataset). To avoid correlations of the response within those localities, each of the 100 bootstrapped samples consisted of a bootstrapped sample of species from localities contributing one time series of abundance *and* one species selected randomly from each of the localities contributing more than one time series of abundance. Herrando-Pérez et al. [20] used 500 and 1,000 bootstrapped repetitions and found similar results and identical ecological interpretation. We measured relative model support across the model set by the medians and 95^th^ percentile ranges of the model probabilities (*w*AIC*_c_*) over all bootstrapped samples. Further, we used model averaging [52] to estimate the coefficient of the effect size of each climate variable on the strength of compensatory density feedback. Thus, we summed (over the 9 models in a set) *w*AIC*_c_* for each model containing a given climate variable weighted by its effect size as a measure of model-averaged effect size. To compare the effect sizes of all explanatory variables (which had disparate units and ranges), we applied a *post hoc* standardization whereby we multiplied model-averaged effect sizes by the product *variable i × sd(response)/sd(variable i)*. We calculated standardized model-averaged effects sizes as their median values and 95^th^ percentile ranges over all bootstrapped samples.

Finally, our previous research has shown the need to control for body size when looking at patterns of change in density feedback across multiple species [20]. A substantial component of the phylogenetic signal in density-feedback strength was attributed to body size. We did also fit generalized linear mixed-effects models (GLMM, [58]) for all taxa, and through generalized linear models (GLM) for birds and mammals separately, that included body size as a fixed effect (Text S2, Table S8 in File S1). However, this approach did not provide any additional information over the PGLS analyses.

In summary, our analyses quantified effect sizes that equate the magnitude of change in density-feedback strength in response to temperature or precipitation, over and above any effects due to time-series length and phylogenetic relatedness among species. We report PGLS results for all species in the main text (Figures 1–3, Tables 1–6). The Supporting Information covers File S1 with PGLS for the high-quality data subsets (Figures S3–S5, Tables S2–S7 in File S1), GLMM and GLM results (Figures S6, S7, Tables S9–S12 in File S1), and additional bibliographic references; and File S2 with the complete dataset.

**Table 4:** Density feedback and mean climate variables.

Variable	birds	mammals
*q*	**–0.04** [–0.05, –0.04]	**–0.07** [–0.09, –0.06]
mT	**-0.04E-3** [-0.07E-3, -0.02E-3]	**-0.02E-2** [-0.04E-2, -0.01E-2]
mP	**0.01** [-0.07E-1, 0.01]	**0.09E-2** [-0.11E-2, 0.35E-2]
sT	**0.03E-6** [0.02E-6, 0.06E-6]	**0.04E-6** [0.03E-6, 0.07E-6]
sP	**0.08E-2** [-0.03E-2, 0.2E-1]	**-0.09E-1** [-0.22E-1, -0.03E-1]

Standardized model-averaged effect sizes of time-series length (*q*, years), mean annual temperature (mT, °C), mean annual precipitation (mP, mm), seasonality of temperature (sT  =  sd, °C) and seasonality of precipitation (sP  =  CV) as explanatory variables of variation in strength of compensatory density feedback in birds (91 species) and mammals (55 species). Statistical models were fitted as phylogenetic generalized least-squares regression, with a total of 9 models in the set (Table 1, Figure 1). Effect sizes are medians (in bold) for 100 bootstrapped samples [95^th^ bootstrapped percentile ranges].

**Table 5:** Density feedback and minimum climate variables.

Variable	birds	mammals
*q*	**–0.05** [–0.05, –0.04]	**–0.07** [–0.09, –0.05]
minT	**-0.03E-3** [-0.04E-3, -0.02E-3]	**-0.04E-3** [-0.07E-3, -0.02E-3]
minP	**0.02E-1** [0.02E-1, 0.03E-1]	**0.04E-2** [-0.01E-2, 0.07E-2]
sT	**0.02E-6** [0.01E-6, 0.04E-6]	**0.06E-6** [0.03E-6, 0.08E-6]
sP	**0.05E-2** [-0.04E-2, 0.02E-1]	**–0.01** [–0.02, -0.04E-1]

Standardized model-averaged effect sizes of time-series length (*q*, years), temperature of the coldest month (minT, °C), precipitation of the driest month (minP, mm), seasonality of temperature (sT  =  sd, °C) and seasonality of precipitation (sP  =  CV) as explanatory variables of variation in strength of compensatory density feedback in birds (91 species) and mammals (55 species). Statistical models were fitted as phylogenetic generalized least-squares regression, with a total of 9 models in each contrasted set (Table 2, Figure 2). Effect sizes are medians (in bold) for 100 bootstrapped samples [95^th^ bootstrapped percentile ranges].

**Table 6:** Density feedback and maximum climate variables.

Variable	birds	mammals
*q*	**–0.04** [–0.05, –0.04]	**–0.06** [–0.08, –0.05]
maxT	**-0.02E-3** [-0.05E-3, -0.03E-4]	**-1.6E-2** [-0.04E-2, -0.06E-3]
maxP	**0.03** [0.02, 0.06]	**-0.05** [-0.09, -0.02]
sT	**0.06E-6** [0.03E-6, 0.01E-5]	**0.07E-6** [0.03E-6, 0.01E-5]
sP	**0.07E-2** [-0.01E-2, 0.02E-1]	**–0.01** [–0.02, -0.03E-1]

Standardized model-averaged effect sizes of time-series length (*q*, years), temperature of the hottest month (maxT, °C), precipitation of the wettest month (maxP, mm), seasonality of temperature (sT  =  sd, °C) and seasonality of precipitation (sP  =  CV) as explanatory variables of variation in strength of compensatory density feedback in birds (91 species) and mammals (55 species). Statistical models were fitted as phylogenetic generalized least-squares regression, with a total of 9 models in each contrasted set (Table 3, Figure 3). Effect sizes are medians (in bold) for 100 bootstrapped samples [95^th^ bootstrapped percentile ranges].

## Results

The strength of compensatory density feedback ranged from –1.55 to –0.01 in birds (median  =  –0.49, 95^th^ percentiles of [–1.21, –0.05]) and from –1.50 to –0.01 in mammals (median  =  –0.32 [–1.23, –0.02]). Between 1950 and 2000, the study localities had mean temperatures that varied from –11.9 to 26.1 °C (median  =  8.8 [–0.9, 18.1]) and from –11.5 to 22.7 °C (median  =  7.0 [–3.6, 22.7]) for birds and mammals, respectively. Considering correlations between climate variables and latitude *or* longitude (Figure S2 in File S1), mean temperature decreased in bird (*ρ*  =  –0.75) and mammal (*ρ*  =  –0.50) localities from low to high latitudes [likewise for temperature of the hottest month in birds (*ρ*  =  –0.66)]. On the other hand, mean precipitation varied from 138 to 2341 mm (median  =  751 [355, 1820]) and from 98 to 2475 mm (median  =  669 [165, 1347]) for bird and mammal localities, respectively. All precipitation variables were poorly correlated with latitude or longitude (Figure S2 in File S1). Our dataset represented the full range of Köppen-Geiger ‘main climates’ worldwide [25], including ‘warm temperate’ (47% of species’ localities), ‘snow’ (35%), ‘arid’ (10%), ‘polar’ (7%) and ‘equatorial’ (< 1%). A total of 13 of the 30 Köppen-Geiger ‘climate types’ were captured, with a predominance of ‘warm temperate’ (31%) and ‘snow’ (17%) both with a fully humid precipitation regime and a warm summer (Table S1 in File S1).

When we contrasted our model sets expressing strength of compensatory density feedback as a function of climate, single models only explained between 1.8 and 3.6 of the median variation (over all bootstrapped samples, see Methods) in feedback strength across birds or mammals (Tables 1–3). Most importantly, the control variable (time-series length) explained most or all of such variation (1.9 to 3.6%). The model including only the control variable (null model) was top-ranked in birds (Figures 1–3) in 69 to 100% of the bootstrapped samples and had 1.1 to 7.7 more support than other models in a set as inferred from evidence ratios of model probabilities (Tables 1–3). For mammals, the model including time-series length and mean annual temperature was top-ranked (Figure 1) in 82% of the bootstrapped samples, and had between 1.9 and 6.5 times more support than other models in the set (Table 1). Therein, the null model was top-ranked in the model sets including minimum and maximum climate variables (Figures 2, 3, Tables 2, 3).

Median model-averaged effect size (standardized over all explanatory variables, see Methods) was highest for the control variable of time-series length in all model sets for birds and mammals (Tables 4–6). All temperature variables had effect sizes near zero. On the other hand, density-feedback strength was negatively correlated with precipitation seasonality in mammals (Figures 1–3, Tables 4–6) and positively correlated with mean precipitation in birds (Figure 1, Table 4). Precipitation of the wettest month had the highest effect size among climate variables, being positive for birds (Table 6) and negative for mammals (Table 6). We replicated our model contrasts for bird and mammal data subsets including high-quality time series, after accounting for stationarity, trending, outliers and missing values (criteria explained in Text S1, File S1). Our results were upheld in that time-series length explained most variation in density-feedback strength over bird and mammal species (Tables S2, S4, S6 in File S1), while effect sizes were of similar magnitude and sign as for the PGLS on the full datasets (Figures S3–S5, Tables S3, S5, S7 in File S1). Model fitting through GLMM and GLM confirmed that the null model (including only the control variables of time-series length and body size) was top-ranked in all model contrasts, and had 19 (all taxa), 10 (birds) and 2 (mammals) times more median information-theoretic support than any second-ranked model including climate variables (Figures S6, S7, Tables S9, S11 in File S1), and the coefficients of all climate predictors were near 0 (Tables S10, S12 in File S1).

## Discussion

We found negligible support for the hypothesis that spatial variation in broad-scale and long-term precipitation and temperature variables can explain spatial variation in strength of compensatory density feedback, based on censuses of 146 species of birds and mammals. We detected effects comparable to those of the control variable (time-series length) for (particularly) precipitation of the wettest month but, given the poor goodness of fit of our models, the biological meaning of those effects can only be suggestive. Our study is the first published quantitative assessment of those correlations, including controls for taxonomic non-independence and allometry, and a quantification of relative effect sizes across more than two taxonomic orders. Our results contrast with the strong correlations reported for ungulates (several populations of two species) in the only cross-taxa study using an analogous modelling framework [19]. Such apparent discrepancy might reflect an interplay between density-dependent and -independent factors at the population level that does not leave a species-specific signal (see below).

In another relevant cross-taxa study, Knape and de Valpine [13] modelled (via autoregression) fluctuations in population size (rather than our metric of density-feedback strength) in response to immediate and delayed density feedback, weather (temperature, precipitation) and climate (North Atlantic and Southern oscillations) for 492 populations of mammals, birds and insects (327 species; J. Knape, pers. comm.). This showed that model-averaged prediction error (of population size from one year to the next) was poorly correlated with latitude – no phylogenetic or allometric control was applied. Again, the lack of pattern of climate signals in population dynamics across species contrasts with unequivocal signals found in some well-studied, single populations in both terrestrial and aquatic ecosystems [2]. For instance, in Soay sheep (*Ovis aries*) at Hirta (St Kilda Archipelago, Scotland), broad climatic indices are robust predictors (better than local weather) of population change because pulses of mortality (mainly by starvation resulting from crowding) consistently occur from January to May every year [59]. The relative effects of climate and density feedback have also been quantified in groups of sympatric species, e.g., large ungulates [60], [61], ducks [62], diurnal or nocturnal butterflies [9], [11], and flatfish [63]. Moreover, latitude has often been used as a proxy for climate, with numerous studies reporting the predominance of immediate versus delayed density feedback (and contrasting dynamics from damping through cycles or chaos) along large latitudinal bands, especially in small rodents, pest insects and game homeotherms (reviewed in [64]). All the latter investigations reveal ample variation in the interplay between climate and density feedbacks at the population level. Therefore, the choice of populations used to represent a species might lead to different results and varying patterns.

A common approach to examine cross-taxa patterns of density feedback, population dynamics and climate is to use a common spatial climate-data resolution for all species, as in our study. This disregards the fact that climate processes driving population change might operate at different spatial scales for different populations and species. For instance, territorial birds can compete for food resources mainly at the scale of territories [65]; the quality of a few plant individuals can override the strength of compensatory density feedback at a population level in herbivorous insects [66]; or, more intricately, the strength of those feedbacks in reef fish can increase at small scales, or decrease at large scales, from low to high habitat complexity [67]. Interestingly, we found that the effect of precipitation of the wettest month on density-feedback strength was positive for birds, and negative for mammals, indicating that both groups might (tentatively) respond differently to broad climatic cues. Although those effects might not be biologically meaningful, given the low goodness of fit of our models, this result alerts that climate effects might cancel out, and so be opaque to modelling, if datasets pool taxa for which density feedbacks vary in opposite direction in response to climate gradients. For future studies over broad taxonomical groups, we suggest the compilation of data from replicate populations for each species, and from species whose demography is known *a priori* to respond to common scales and cues of environmental variation, such as in territorial, long-distance migratory or small oceanic-island species – this enterprise might require collaborative effort among many researchers sharing their data on individual populations, or access to data from national environmental agencies monitoring populations and species for decades [68]). Allometric and/or phylogenetic controls are also indispensable in cross-taxa comparisons as supported by our results. Critically, those results were upheld by applying phylogenetic constraints (PGLS based on fine phylogenies of birds and mammals) and simpler linear models (GLM, and GLMM with taxonomic *Order* as random factor, both with controls for body size); therefore, cross-taxa analyses can be feasible for those groups of species for which robust phylogenies might be unavailable.

A fundamental limitation of our theme of investigation is that ‘temperate’ and ‘snow’ predominated relative to ‘equatorial’, ‘arid’ and ‘polar’ climates. This bias originates from the state of the art of ecological research that concentrates on temperate climates in wealthy countries mostly in the Northern Hemisphere [69] – a trend particularly pronounced in the study of density feedbacks across taxa and biomes [70]. Indeed, the length of the climate gradients captured by our climate variables might be too narrow to signal spatial variation in the strength of density feedback; in particular, the paucity of demographic data from tropical populations certainly truncates the full spectrum of variation for both temperature and precipitation variables and reduces the power for testing our hypothesis relative to other macroecological studies (e.g., [71]). At present, this caveat could be partly superseded by combing the literature for long-term population data from poorly studied climates and biomes. Much of this information might be available in grey literature or environmental reports, or awaits collection in future research.

In the last two decades, new developments in mathematical demography have shifted the focus from *testing for* (the presence of) to *explaining variation in* density feedback [3]. We have cited above a sample of a large body of recently published studies aiming to elucidate the relative demographic role of exogenous and endogenous mechanisms. Meta-analytical techniques hold a promising future application here (e.g., [72]), but care must be taken to ensure parameter estimates and hypotheses are comparable across species, statistical models and studies. For instance, the estimation of additive effects of autoregressive parameters of climate/weather and lagged population size is often used to explain or predict temporal change in a range of different responses, such as process error [13], population size [38] or population growth rate [73]. For example, Post [61] used time series of the North Atlantic Oscillation (NAO) and times series of abundance of 27 populations of caribou/reindeer (*Rangifer tarandus*) in Greenland, Finland and Russia in an autoregressive model (response  =  *N_t_*, explanatory variables  =  density feedback and climate). He estimated effect sizes of density feedback and NAO, and subsequently correlated those effect sizes (now functioning as responses) with latitude and longitude (new explanatory variables) – see also our description of Knape and de Valpine’s [13] response and explanatory variables above. In contrast, in our study and that of Wang et al. [19], time series of abundance of birds and mammals have been used to estimate the strength of compensatory density feedback (response  =  *r*, explanatory variable  =  *N_t_*), and subsequently, we correlated feedback strength (new response) with single, average climate variables. In doing so, our study examined how the intensity of trophic/social interactions (as inferred from density feedback) across species can vary with long-term average external forcing. Thus, the selection of different responses and potential explanatory variables, and of different model sets and modelling approaches, potentially addresses the interplay between climate and density feedbacks from different angles, but comes at the expense of ease of comparability.

## Further Directions

Mechanistic understanding lags behind mathematical development and model fitting in ecology and such a mismatch has handicapped the identification of ‘general principles’ in population dynamics [74]. Among those developments, time-series analyses have become an important tool in macroecological research [2], [3], [75], and shed light on important fundamental themes such as the relationship between demography and life history [76], evolution [77] or extinction [78], often using datasets spanning invertebrates, vertebrates and plants from aquatic and terrestrial realms. Yet, the disparity of the spatial scales, at which environmental forcing might affect the population dynamics of species with contrasting life-history traits (e.g., body size, fertility, longevity, age at first reproduction), mobility and habitat dependencies, suggests that the study of the interplay of density-independent and -dependent factors through time-series analysis might only be biologically meaningful (and result in some general cross-taxa patterns) among closely related species, whereby long-term data include several population per species to account for intraspecific variation. We strongly argue that our ability to discern global patterns of population dynamics is currently limited because parameters, models and modelling approaches (hence underlying hypotheses) are not directly comparable across studies. Furthermore, long-term studies based on census data and summary statistics of reproductive fitness (like *r*) are largely opaque in identifying the actual mechanisms causing demographic feedbacks [79]; for that, experimentation is likely to be more appropriate [80]. Indeed, the conceptual rationale of our study stems from Nicholson’s iconic experiments on blowflies (*Lucilia cuprina*), where he hypothesized that intraspecific competition drove oscillations in numbers of larvae and adults exposed to different amounts of food resources [81], [82]. Surprisingly, this kind of experimentation has received little attention thereafter, and the results from already published experiments still await meta-analytical enquiry (e.g., [83]–[86]). Along with a more unified theoretical framework, whereby hypotheses (rather than statistics) drive research progress [74] and researchers communicate more effectively [87], the connection between long-term/large-scale and short-term/small-scale studies, presently confined to focal taxa and specialities (e.g., [72], [88]–[90]), seems crucial to improve our mechanistic understanding of population dynamics.

## Supporting Information

File S1 **Supporting information file including Texts S1, S2, Tables S1-S12, and Figures S1-S8. Text S1, Criteria for high-quality data subsets.** **Text S2, Dataset properties, modelling approach and results for GLM/GLMM analyses. Table S1, Frequency of Köppen-Geiger climate types captured by the full dataset and the high-quality subsets.** **Table S2, Density feedback and mean climate variables for high-quality data subsets.** Akaike’s information criterion (support for the model set correlating temperature and precipitation variables to strength of compensatory density feedback for birds and mammals. All models were fitted through phylogenetic generalized least-squares regression, and model-ranking descriptors are medians from 100 bootstrapped samples. **Table S3, Density feedback and mean climate variables for high-quality data subsets.** Standardized model-averaged effect sizes of time-series length, mean annual temperature, mean annual precipitation, seasonality of temperature and seasonality of precipitation as explanatory variables of variation in strength of compensatory density feedback in birds or mammals. Statistical models were fitted as phylogenetic generalized least-squares regression, with a total of nine models in each contrasted set. Effect sizes are medians for 100 bootstrapped samples. **Table S4, Density feedback and minimum climate variables for high-quality data subsets.** Akaike’s information criterion support for the model set correlating temperature and precipitation variables to strength of compensatory density feedback for birds and mammals. All models were fitted using phylogenetic generalized least-squares regression, and model-ranking descriptors are medians from 100 bootstrapped samples. **Table S5, Density feedback and minimum climate variables for high-quality data subsets.** Standardized model-averaged effect sizes of time-series length, temperature of the coldest month, precipitation of the driest month, seasonality of temperature and seasonality of precipitation as explanatory variables of variation in strength of compensatory density feedback in birds or mammals. Statistical models were fitted as phylogenetic generalized least-squares regression, with a total of nine models in each contrasted set. Effect sizes are medians for 100 bootstrapped samples. **Table S6, Density feedback and maximum climate variables for high-quality data subsets.** Akaike’s information criterion support for the model set correlating temperature and precipitation variables1 to strength of compensatory density feedback for birds and mammals. All models were fitted using phylogenetic generalized least-squares regression, and model-ranking descriptors are medians from 100 bootstrapped samples. **Table S7, Density feedback and maximum climate variables for high-quality data subsets.** Standardized model-averaged effect sizes of time-series length, mean temperature of the hottest month, mean precipitation of the wettest month, seasonality of temperature and seasonality of precipitation as explanatory variables of variation in strength of compensatory density feedback in birds or mammals. Statistical models were fitted as phylogenetic generalized least-squares regressions, with a total of nine models in each contrasted set. Effect sizes are medians for 100 bootstrapped samples. **Table S8, Model set used for GLMM and GLM analyses.** All models include the same response, i.e., strength of compensatory density feedback across taxa (fitted by GLMM and OR  =  taxonomic *order* as random factor), and bird and mammal species (fitted by GLM, no phylogenetic random effect). Control variables were included in all models, namely *q*  =  length of time series, and *body*  =  body size. Climate variables encompassed: mT  =  annual temperature, mP  =  annual precipitation, sT  =  seasonality of temperature, and sP  =  seasonality of precipitation. **Table S9, Density feedback, mean climate variables and model contrasts using linear models.** Akaike’s information criterion support for the first- and second-ranked models correlating temperature and precipitation variables to strength of compensatory density feedback for all taxa, and only mammals or birds. All models were fitted under a generalized linear mixed-effects framework for all taxa and included three control variables, namely time-series length, body size and the taxonomic level of *order* as random factor. We used generalized linear models (i.e., without a random factor) for the data subsets of birds and mammals separately. Model-ranking descriptors are medians from 100 bootstrapped samples. **Table S10, Density feedback, mean climate variables and model contrasts using linear models.** Standardized model-averaged effect sizes of time-series length, body size, mean annual temperature, mean annual precipitation, seasonality of temperature and seasonality of precipitation as explanatory variables of variation in strength of compensatory density feedback, for all taxa, and only mammals or birds. Statistical models were fitted as generalized linear mixed effects (all taxa) or generalized linear models (birds or mammals, separately), with a total of 9 models in each contrasted set. Effect sizes are medians for 100 bootstrapped samples. **Table S11, Density feedback, mean climate variables and model contrasts using linear models for the high-quality data subsets.** Akaike’s information criterion support for the first- and second-ranked models correlating temperature and precipitation variables to strength of compensatory density feedback for all taxa, and only mammals or birds. All models were fitted under a generalized linear mixed effects framework for all taxa and included three control variables, namely time-series length, body size and the taxonomic level of *order* as random factor. We used generalized linear models (i.e., without a random factor) for the data subsets of birds and mammals separately. Model-ranking descriptors are medians from 100 bootstrapped samples. **Table S12, Density feedback, mean climate variables and model contrasts using linear models for the high-quality data subsets.** Standardized model-averaged effect sizes of time-series length, body size, mean annual temperature, mean annual precipitation, seasonality of temperature and seasonality of precipitation as explanatory variables of variation in strength of compensatory density feedback, for all taxa, and only mammals or birds. Statistical models were fitted as generalized linear mixed effects (all taxa) or generalized linear models (birds or mammals, separately), with a total of nine models in each contrasted set. Effect sizes are medians for 100 bootstrapped samples. **Figure S1, Map of localities.** Position of the 97 study localities (28 countries) over 146 species of birds and mammals covered in the phylogenetic generalized least-squares regression for all species, and the high-quality subset including 76 birds and 44 mammals from 94 localities and 26 countries. **Figure S2, Bivariate correlations among climate variables, latitude and longitude.** Spearman correlations and bivariate plots among latitude, longitude and the climate variables, namely average temperature, temperature seasonality, minimum temperature in the coldest month, maximum temperature in hottest month, average precipitation, precipitation seasonality, minimum precipitation in driest month, and maximum precipitation in the wettest month. Taxa comprise 91 bird and 55 mammal species from 97 localities covered in the phylogenetic generalized least-squares regressions. Latitude and longitude are absolute values so representing positions from the equator to the poles in both hemispheres. **Figure S3, Density feedback and mean climate variables for high-quality data subsets.** Model probabilities and standardized wAIC*_c_*-averaged effect sizes after contrasting 9 models with strength of compensatory density feedback from time series of abundance and combinations of six explanatory variables including time-series length, mean annual temperature, mean annual precipitation, seasonality of temperature and seasonality of precipitation. Models were fitted as phylogenetic generalized least-squares regressions for two datasets comprising 77 bird and 45 mammal species, respectively. **Figure S4, Density feedback and minimum climate variables for high-quality data subsets.** Model probabilities and standardized wAIC*_c_*-averaged effect sizes after contrasting nine models with strength of compensatory density feedback from time series of abundance and combinations of six explanatory variables including time-series length, temperature of the coldest month, precipitation of the driest month, seasonality of temperature and seasonality of precipitation. Models were fitted as phylogenetic generalized least-squares regressionsfor two datasets comprising 76 bird and 44 mammal species, respectively. **Figure S5, Density feedback and maximum climate variables for high-quality data subsets.** Model probabilities and standardized wAIC*_c_*-averaged effect sizes after contrasting nine models with strength of compensatory density feedback from time series of abundance and combinations of 6 explanatory variables including time-series length, temperature of the hottest month, precipitation of the wettest month, seasonality of temperature and seasonality of precipitation. Models were fitted as phylogenetic generalized least-squares regressions for two datasets comprising 91 bird and 55 mammal species, respectively. **Figure S6, Density feedback, mean climate variables and model contrasts using linear models.** Model probabilities and standardized AIC*_c_*-averaged effect sizes after contrasting 9 models with strength of compensatory density feedback from time series of abundance and combinations of 6 explanatory variables including time-series length, body size, mean annual temperature, mean annual precipitation, seasonality of temperature and seasonality of precipitation. Statistical models were fitted as generalized linear mixed-effects models (random effect  =  Linnean taxonomical *order*) for all taxa and as generalized linear models for the subsets of birds and mammals. **Figure S7, Density feedback, mean climate variables and model contrasts using linear models for the high-quality data subsets.** Model probabilities and standardized AIC*_c_*-averaged effect sizes after contrasting 9 models with strength of compensatory density feedback from time series of abundance and combinations of 6 explanatory variables including time-series length, body size, mean annual temperature, mean annual precipitation, seasonality of temperature and seasonality of precipitation. Statistical models were fitted as generalized linear-mixed effects models (random effect  =  Linnaean taxonomical *order*) for all taxa and as generalized linear models for the subsets of birds and mammals.
(DOCX)

File S2 **Supporting Dataset.**
(XLS)
