# Supplementary material for: Spatial Climate Patterns Explain Negligible Variation in Strength of Compensatory Density Feedbacks in Birds and Mammals
Source: PLoS One. 2014 Mar 11;9(3):e91536. doi: 10.1371/journal.pone.0091536 (PMC3950218; doi:10.1371/journal.pone.0091536)
Supplement: File S1 — Supporting information file including Texts S1, S2, Tables S1-S12, and Figures S1-S8. Text S1, Criteria for high-quality data subsets. Text S2, Dataset properties, modelling approach and results for GLM/GLMM analyses. Table S1, Frequency of Köppen-Geiger climate types captured by the full dataset and the high-quality subsets. Table S2, Density feedback and mean climate variables for high-quality data subsets. Akaike’s information criterion (support for the model set correlating temperature and precipitation variables to strength of compensatory density feedback for birds and mammals. All models were fitted through phylogenetic generalized least-squares regression, and model-ranking descriptors are medians from 100 bootstrapped samples. Table S3, Density feedback and mean climate variables for high-quality data subsets. Standardized model-averaged effect sizes of time-series length, mean annual temperature, mean annual precipitation, seasonality of temperature and seasonality of precipitation as explanatory variables of variation in strength of compensatory density feedback in birds or mammals. Statistical models were fitted as phylogenetic generalized least-squares regression, with a total of nine models in each contrasted set. Effect sizes are medians for 100 bootstrapped samples. Table S4, Density feedback and minimum climate variables for high-quality data subsets. Akaike’s information criterion support for the model set correlating temperature and precipitation variables to strength of compensatory density feedback for birds and mammals. All models were fitted using phylogenetic generalized least-squares regression, and model-ranking descriptors are medians from 100 bootstrapped samples. Table S5, Density feedback and minimum climate variables for high-quality data subsets. Standardized model-averaged effect sizes of time-series length, temperature of the coldest month, precipitation of the driest month, seasonality of temperature and seasonality of precipitatio [file pone.0091536.s001.docx]

**SUPPORTING INFORMATION**

**Herrando-Pérez, Delean, Brook, Cassey and Bradshaw**

Spatial climate patterns explain negligible variation in strength of compensatory density feedbacks in birds and mammals

***Index***

- Map of localities (Figure S1)
- Bivariate correlations among climate variables, latitude and longitude (Figure S2)
- Frequency of Köppen-Geiger climates (Table S1)
- PGLS model rankings and effect sizes for high-quality data subsets, including data-selection criteria (Figures S3 to S6, Tables S2 to S7), including (Text S2) criteria of selection of time series
- Generalized linear (with/without mixed-effects) models (Figures S7, S8, Tables S8 to S12), including (Text S2) properties of the dataset, modelling approach and results
- References

**Figure S1. Map of localities.**

Position of the 97 study localities (28 countries) over 146 species of birds (*N* = 91) and mammals (*N* = 55) covered in the phylogenetic generalized least-squares regression for all species, and the high-quality (hq) subset including 76 birds and 44 mammals from 94 localities and 26 countries.


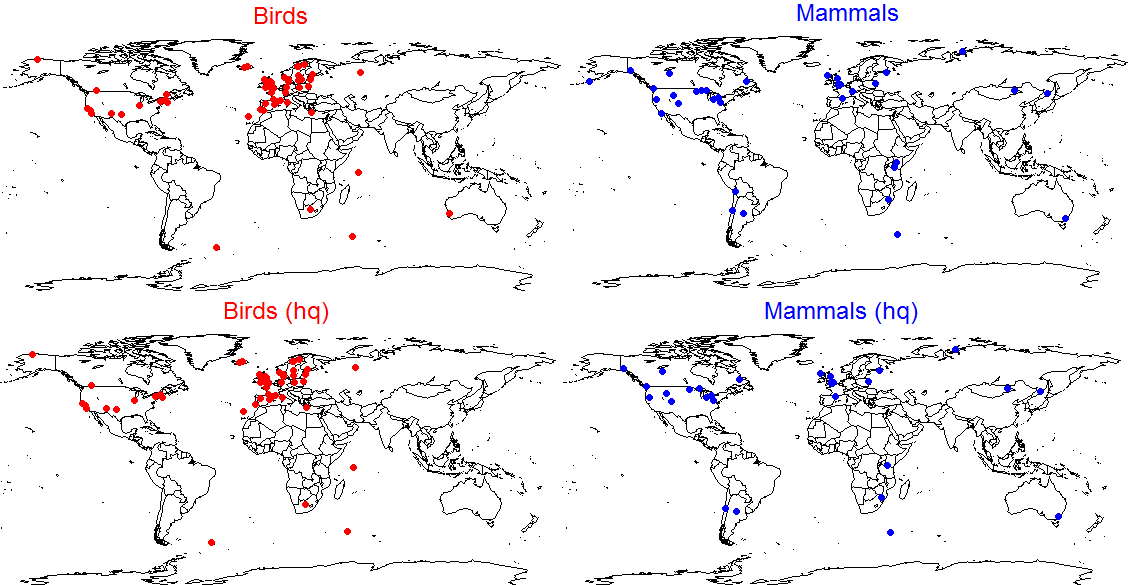


**Figure S2. Bivariate correlations among climate variables, latitude and longitude.**

Spearman correlations (upper panels where number increases with correlation magnitude) and bivariate plots (lower panels) among latitude, longitude and the climate variables, namely average temperature (mT,°C), temperature seasonality (sT = sd,°C), minimum temperature in the coldest month (minT, °C), maximum temperature in hottest month (maxT, °C), average precipitation (log(mP), mm), precipitation seasonality (log(sP)) = CV, minimum precipitation in driest month (log(minP)), and maximum precipitation in the wettest month (log(maxP)). Taxa comprise 91 bird (top graph) and 55 mammal (bottom graph) species from 97 localities covered in the phylogenetic generalized least-squares regressions (PGLS). Latitude and longitude are absolute values so representing positions from the equator to the poles in both hemispheres.

**
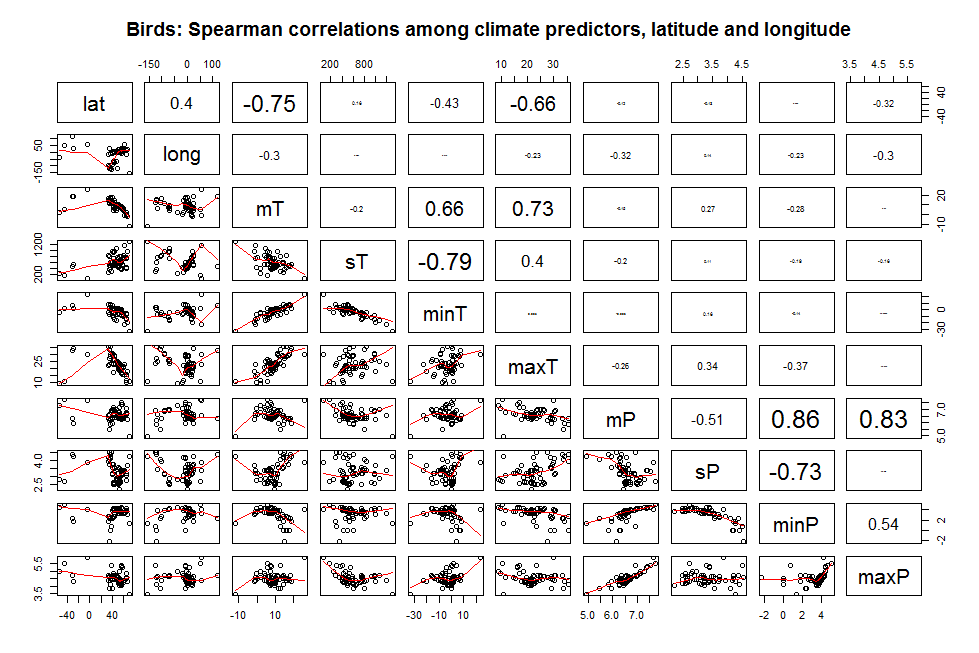

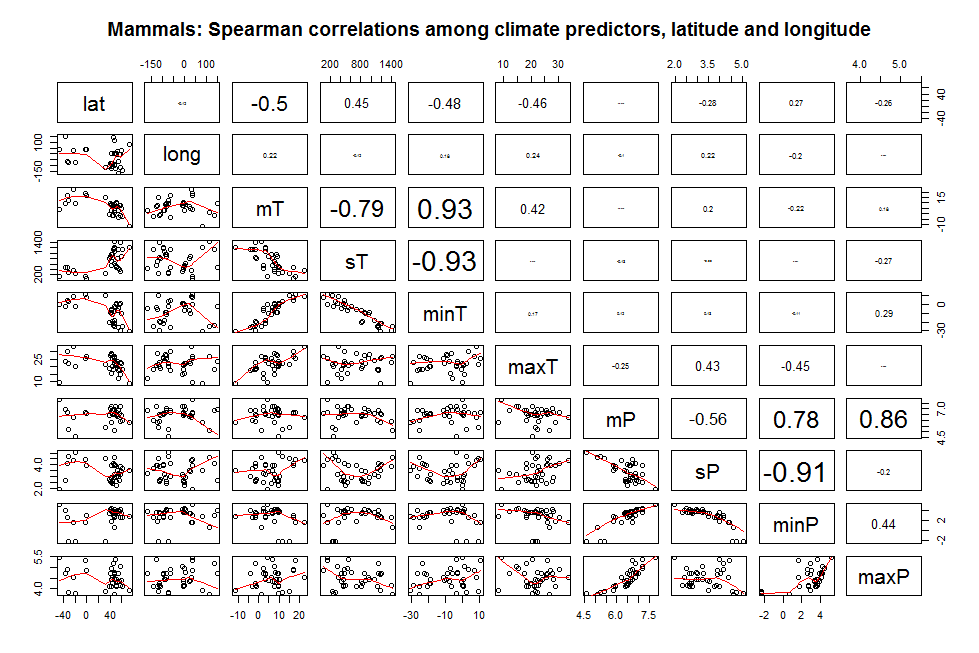
**

**Frequency of Köppen-Geiger climates**

**Table S1. Frequency of Köppen-Geiger climate types captured by the full dataset (97 localities for 91 bird and 55 mammal species) and the high-quality (hq) subsets (94 localities for 76 bird and 44 mammal species).**

For acronyms, see legend below.

| **Climate** | **Birds** | **Mammals** | **Birds (hq)** | **Mammals (hq)** |
| --- | --- | --- | --- | --- |
| **Af** | 1 | 0 | 1 | 0 |
| **Bsh** | 1 | 4 | 1 | 4 |
| **Bsk** | 2 | 7 | 2 | 6 |
| **BWk** | 0 | 1 | 0 | 0 |
| **Cfa** | 1 | 1 | 1 | 1 |
| **Cfb** | 33 | 10 | 29 | 9 |
| **Csa** | 8 | 0 | 4 | 0 |
| **Csb** | 5 | 2 | 5 | 1 |
| **Cwb** | 0 | 3 | 0 | 3 |
| **Dfa** | 9 | 1 | 4 | 1 |
| **Dfb** | 14 | 16 | 12 | 11 |
| **Dfc** | 10 | 6 | 10 | 5 |
| **ET** | 7 | 4 | 7 | 3 |

**Legend:** Köppen-Geiger climates are categorized by case-sensitive letters representing descriptors of main climate + precipitation + temperature, in that order. For instance. ‘Cfb’ stands for ‘warm temperate’ main climate (C), with a ‘fully humid’ precipitation regime (f) and a ‘warm summer’ (b). There are a total of 5 main climates and 30 climate types under this classification. See acronyms below and further details in [[1](#_ENREF_1),[2](#_ENREF_2)].

| **ACRONYMS** |  |  |  |  | **CATEGORIES** |  |
| --- | --- | --- | --- | --- | --- | --- |
| **Main climate** | **Precipitation** | **Temperature** |  |  | **Main climates** | **Types** |
| A: equatorial | W: desert | h: hot arid |  |  | A | Af, Am, As, Aw |
| B: arid | S: steppe | k: cold arid |  |  | B | BWk, BWh, BSk, BSh |
| C: warm temperate | f: fully humid | a: hot summer |  |  | C | Cfa, Cfb, Cfc, Csa, Csb, Csc, Cwa, Cwb, Cwc |
| D: snow | s: summer dry | b: warm summer |  |  | D | Dfa, Dfb, Dfc, Dfd, Dsa, Dsb, Dsc, Dsd, Dwa, Dwb, Dwc, Dwd |
| E: polar | w: winter dry | c: cool summer |  |  | E | EF, ET |
|  | m: monsoonal | d: extremely continental |  |  |  |  |
|  | m: monsoonal | F: polar frost |  |  |  |  |
|  |  | T: polar tundra |  |  |  |  |

**PGLS model rankings and effect sizes for high-quality data subsets, including (Text S1) criteria of selection of time series**

**Text S1. Criteria for High-quality Data Subsets.**

We replicated all analyses for the total sample of 146 species (see main text), and a subset of 120 species (76 birds, 44 mammals) from 94 localities in 26 countries (Figure S1) after meeting stringent criteria following Herrando-Pérez et al. [[3](#_ENREF_3)]: stationarity, trending, outliers and missing values. Stationarity implies that endogenous processes driving population dynamics, such as density feedback, vary around means with constant variance and no trending [[4](#_ENREF_4)] ⎯ these properties allows reliable reconstruction of those processes through density-dependent models fitted to time series [[4](#_ENREF_4),[5](#_ENREF_5)]. Given that the degree of stationarity and trending across our sample of time series varied from low to high, we used a conservative (75%) cut-off to exclude time series with moderate-to-strong departures from model assumptions (see below). We first assessed stationarity through the ‘return rate’, i.e. “the time it takes [for a population] to return to equilibrium following disturbance” [[6](#_ENREF_6)] in log*_e_*(population size)-time space, and excluded time series with a coefficient of variation of the return rate (> 4.1) above the 75^th^ percentile. We left out time series for which linear trending of log-transformed population sizes had more statistical support (based on AIC*_c_*) than an intercept-only model *and* slopes > |0.1| (< 25^th^ and > 75^th^ percentiles). We further removed time series where missing-value frequency was ≥ 20 % of the time-series length. We detected time series with extreme outliers through the studentization of the residuals [[7](#_ENREF_7)] from the model supporting linear trending (see above), and removed those with residuals above the 75^th^ percentile (> 3.3). AIC*_c_* metrics from the PGLS on the high-quality data subsets of birds and mammals (separately) are presented in Figures S3 to S5 and Tables S2 to S7, although we summarize those results in the main text.

**Figure S3. Density feedback and mean climate variables for high-quality data subsets.**

Model probabilities (left panels; Table S2) and standardized *w*AIC*_c_*-averaged effect sizes (right panels; Table S3) after contrasting 9 models with strength of compensatory density feedback from time series of abundance (response) and combinations of six explanatory variables including time-series length (*q*, years), mean annual temperature (mT, °C), mean annual precipitation (mP, mm), seasonality of temperature (sT = sd, °C) and seasonality of precipitation (sP = CV). Models were fitted as phylogenetic generalized least-squares regressions for two datasets comprising 77 bird and 45 mammal species, respectively.


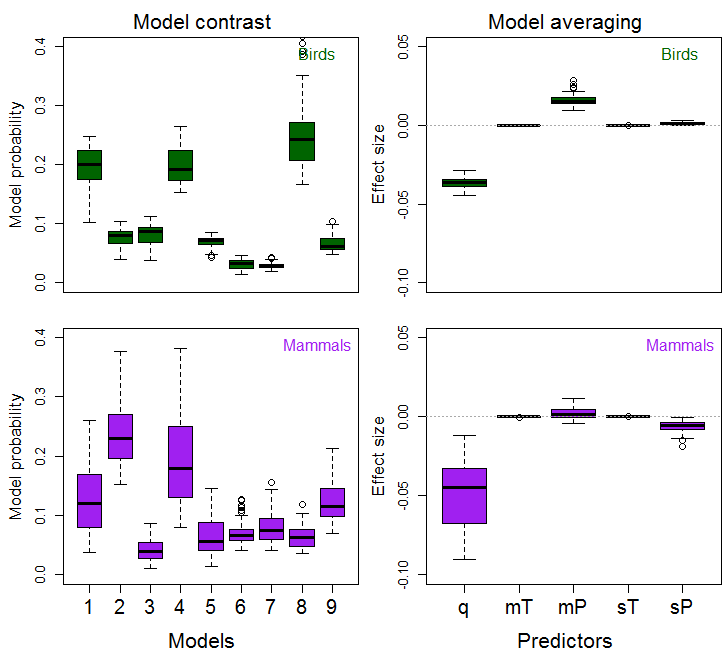


**Table S2. Density feedback and mean climate variables for high-quality data subsets.**

Akaike’s information criterion (AIC*_c_*) support for the model set correlating temperature and precipitation variables^1^ to strength of compensatory density feedback for birds (77 species) and mammals (45 species) (Figure S3). All models were fitted through phylogenetic generalized least-squares regression, and model-ranking descriptors (*w*AIC*_c_*, %variance and *ER*)^2^ are medians from 100 bootstrapped samples [95^th^ percentile ranges].

| **taxa** | **top-ranked models** | ***w*AIC*_c_*** | **%variance** | ***ER*** | **top rank** |
| --- | --- | --- | --- | --- | --- |
| birds | Strength ~ *q* | **0.20** [0.12, 0.24] | **2.9** [2.4, 3.4] | 1.2 | **29** (29) |
|  | Strength ~ *q* + mT | **0.08** [0.05, 0.10] | **2.9** [2.4, 3.3] | 3.0 | **0** (0) |
|  | Strength ~ *q* + mP | **0.09** [0.05, 0.11] | **2.9** [2.4, 3.3] | 2.9 | **0** (0) |
|  | Strength ~ *q* + sT | **0.19** [0.16, 0.26] | **2.8** [2.3, 3.2] | 1.2 | **3** (47) |
|  | Strength ~ *q* + sP | **0.07** [0.05, 0.08] | **2.9** [2.4, 3.3] | 3.5 | **0** (0) |
|  | Strength ~ *q* + mT + mP | **0.03** [0.02, 0.04] | **2.9** [2.4, 3.3] | 7.3 | **0** (0) |
|  | Strength ~ *q* + mT + sP | **0.03** [0.02, 0.04] | **2.9** [2.4, 3.3] | 8.4 | **0** (0) |
|  | Strength ~ *q* + sT + mP | **0.24** [0.18, 0.39] | **2.7** [2.2, 3.1] | - | **68** (24) |
|  | Strength ~ *q* + sT + sP | **0.06** [0.05, 0.09] | **2.8** [2.3, 3.2] | 3.8 | **0** (0) |
| mammals | Strength ~ *q* | **0.12** [0.05, 0.24] | **1.8** [1.3, 2.2] | 2.1 | **9** (19) |
|  | Strength ~ *q* + mT | **0.23** [0.16, 0.36] | **1.4** [1.1, 1.7] | - | **56** (35) |
|  | Strength ~ *q* + mP | **0.04** [0.02, 0.08] | **1.8** [1.2, 2.2] | 6.1 | **0** (0) |
|  | Strength ~ *q* + sT | **0.18** [0.09, 0.35] | **1.4** [1.0, 1.8] | 1.3 | **34** (28) |
|  | Strength ~ *q* + sP | **0.06** [0.02, 0.14] | **1.7** [1.2, 2.0] | 4.0 | **0** (0) |
|  | Strength ~ *q* + mT + mP | **0.07** [0.04, 0.12] | **1.5** [1.1, 1.8] | 3.6 | **0** (2) |
|  | Strength ~ *q* + mT + sP | **0.07** [0.04, 0.13] | **1.4** [1.1, 1.7] | 3.3 | **0** (4) |
|  | Strength ~ *q* + sT + mP | **0.06** [0.04, 0.10] | **1.4** [1.0, 1.9] | 3.6 | **0** (0) |
|  | Strength ~ *q* + sT + sP | **0.12** [0.08, 0.20] | **1.3** [1.0, 1.7] | 1.9 | **1** (12) |

**^1^ mT** = mean annual temperature (°C), **mP =** mean annual precipitation (mm); **sT =** seasonality of temperature (sd, °C), and **sP =** seasonality of precipitation (CV). The model set equated *q* as control variable (i.e., present in all models), eight combinations of climate variables [mT | mP | sT | sP | mT+mP | mT+sP | sT+mP | sT+sP], and a null model with only *q*.

**^2^ *w*AIC*_c_*** = model probabilities given each dataset and model set; %**variance** = % variance in density-feedback strength explained by each model within the set; ***ER*** = evidence ratio of first model over the remaining models according to *w*AIC*_c_*; and **top rank** = times each model was top-ranked over the 100 bootstrapped samples (times each model was second-ranked).

**Table S3. Density feedback and mean climate variables for high-quality data subsets.**

Standardized model-averaged effect sizes of time-series length (*q*, years), mean annual temperature (mT, °C), mean annual precipitation (mP, mm), seasonality of temperature (sT = sd, °C) and seasonality of precipitation (sP = CV) as explanatory variables of variation in strength of compensatory density feedback (response) in birds (77 species) or mammals (45 species). Statistical models were fitted as phylogenetic generalized least-squares regression, with a total of nine models in each contrasted set (Table S2, Figure S3). Effect sizes are medians (in bold) for 100 bootstrapped samples [95^th^ bootstrapped percentile ranges].

| **variable** | **birds** | **mammals** |
| --- | --- | --- |
| *q* | **-0.04** [-0.04, -0.03] | **-0.04** [-0.08, -0.02] |
| mT | **-0.02E-3** [-0.04E-3, -0.08E-4] | **-0.01E-2** [-0.03E-2, -0.08E-3] |
| mP | **0.02** [0.01, 0.02] | **0.02 E-1** [-0.03E-1, 0.01] |
| sT | **0.01E-5** [0.09E-6, 0.02E-5] | **0.07E-6** [0.03E-6, 0.01E-5] |
| sP | **0.01E-1** [0.02E-2, 0.03E-1] | **-0.01** [-0.01,-0.01E-1] |

**Figure S4. Density feedback and minimum climate variables for high-quality data subsets.**

Model probabilities (left panels, Table S4) and standardized *w*AIC*_c_*-averaged effect sizes (right panels; Table S5) after contrasting nine models with strength of compensatory density feedback from time series of abundance and combinations of six explanatory variables including time-series length (*q*, years), temperature of the coldest month (minT, °C), precipitation of the driest month (minP, mm), seasonality of temperature (sT = sd, °C) and seasonality of precipitation (sP = CV). Models were fitted as phylogenetic generalized least-squares regressions for two datasets comprising 76 bird and 44 mammal species, respectively.

**
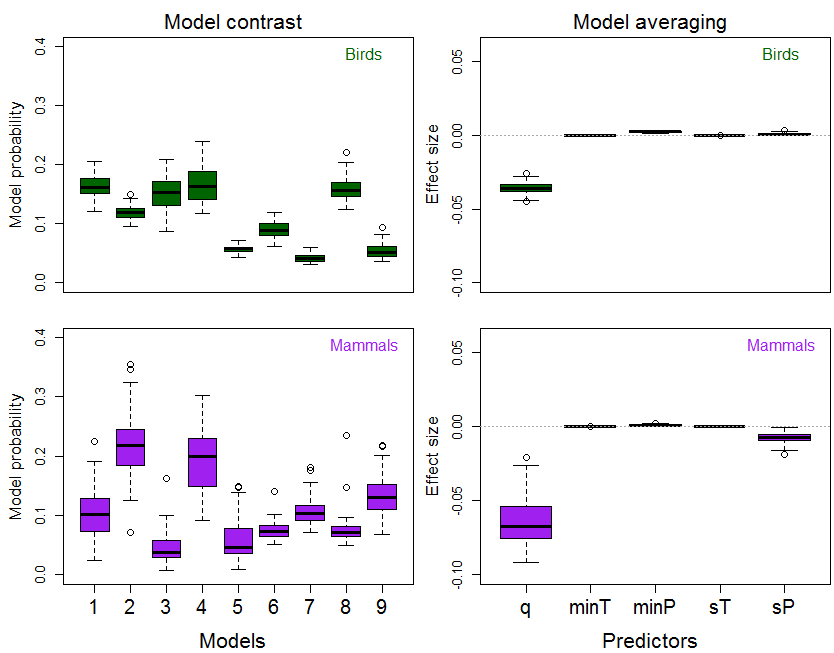
**

**Table S4. Density feedback and minimum climate variables for high-quality data subsets.**

Akaike’s information criterion (AIC*_c_*) support for the model set correlating temperature and precipitation variables^1^ to strength of compensatory density feedback for birds (76 species) and mammals (44 species) (Figure S4). All models were fitted using phylogenetic generalized least-squares regression, and model-ranking descriptors (*w*AIC*_c_*, %variance and *ER*)^2^ are medians from 100 bootstrapped samples [95^th^ percentile ranges].

| **taxa** | **top-ranked models** | ***w*AIC*_c_*** | **%variance** | ***ER*** | **top rank** |
| --- | --- | --- | --- | --- | --- |
| birds | Strength ~ *q* | **0.16** [0.13, 0.20] | **2.9** [2.4, 3.2] | - | **27** (31) |
|  | Strength ~ *q* + minT | **0.12** [0.10, 0.14] | **2.8** [2.4, 3.1] | 1.4 | **0** (1) |
|  | Strength ~ *q* + minP | **0.15** [0.10, 0.19] | **2.8** [2.3, 3.1] | 1.1 | **22** (20) |
|  | Strength ~ *q* + sT | **0.16** [0.12, 0.23] | **2.8** [2.3, 3.1] | 1.0 | **37** (19) |
|  | Strength ~ *q* + sP | **0.06** [0.05, 0.07] | **2.9** [2.4, 3.2] | 2.9 | **0** (0) |
|  | Strength ~ *q* + minT + minP | **0.09** [0.06, 0.12] | **2.7** [2.3, 3.0] | 1.8 | **0** (0) |
|  | Strength ~ *q* + minT + sP | **0.04** [0.03, 0.05] | **2.8** [2.4, 3.1] | 4.0 | **0** (0) |
|  | Strength ~ *q* + sT + minP | **0.16** [0.13, 0.20] | **2.7** [2.2, 3.0] | 1.0 | **14** (29) |
|  | Strength ~ *q* + sT + sP | **0.05** [0.04, 0.08] | **2.8** [2.3, 3.1] | 3.2 | **0** (0) |
|  |  |  |  |  |  |
| mammals | Strength ~ *q* | **0.10** [0.03, 0.17] | **1.6** [1.3, 2.0] | 2.0 | **4** (11) |
|  | Strength ~ *q* + minT | **0.22** [0.13, 0.32] | **1.2** [1.0, 1.7] | 1.0 | **66** (25) |
|  | Strength ~ *q* + minP | **0.04** [0.01, 0.10] | **1.6** [1.3, 2.0] | 5.4 | **0** (0) |
|  | Strength ~ *q* + sT | **0.20** [0.10, 0.26] | **1.2** [1.0, 1.7] | 1.2 | **25** (50) |
|  | Strength ~ *q* + sP | **0.05** [0.01, 0.14] | **1.5** [1.2, 1.8] | 4.7 | **1** (2) |
|  | Strength ~ *q* + minT + minP | **0.07** [0.06, 0.10] | **1.2** [1.0, 1.7] | 3.1 | **0** (0) |
|  | Strength ~ *q* + minT + sP | **0.10** [0.07, 0.15] | **1.2** [1.0, 1.6] | 2.2 | **0** (4) |
|  | Strength ~ *q* + sT + minP | **0.07** [0.06, 0.10] | **1.2** [1.0, 1.7] | 2.9 | **0** (1) |
|  | Strength ~ *q* + sT + sP | **0.13** [0.08, 0.20] | **1.2** [1.0, 1.5] | 1.5 | **4** (7) |

**^1^ minT** = temperature of the coldest month (°C), **minP =** precipitation of the driest month (mm); **sT =** seasonality of temperature (sd, °C), and **sP =** seasonality of precipitation (CV). The model set equated *q* as control variable (i.e., present in all models), eight combinations of climate variables [minT | minP | sT | sP | minT+minP | minT+sP | sT+minP | sT+sP], and a null model with only *q*.

**^2^ *w*AIC*_c_*** = model probabilities given each dataset and model set; %**variance** = % variance in density-feedback strength explained by each model within the set; ***ER*** = evidence ratio of first model over the remaining models according to *w*AIC*_c_*; and **top rank** = times each model was top-ranked over the 100 bootstrapped samples (times each model was second-ranked).

**Table S5. Density feedback and minimum climate variables for high-quality data subsets.**

Standardized model-averaged effect sizes of time-series length (*q*, years), temperature of the coldest month (minT, °C), precipitation of the driest month (minP, mm), seasonality of temperature (sT = sd, °C) and seasonality of precipitation (sP = CV) as explanatory variables of variation in strength of compensatory density feedback in birds (76 species) or mammals (44 species). Statistical models were fitted as phylogenetic generalized least-squares regression, with a total of nine models in each contrasted set (Table S4, Figure S4). Effect sizes are medians (in bold) for 100 bootstrapped samples [95^th^ bootstrapped percentile ranges].

| **variable** | **birds** | **mammals** |
| --- | --- | --- |
| *q* | **-0.04** [-0.04, -0.03] | **-0.05** [-0.08, -0.01] |
| minT | **-0.03E-3** [-0.05E-3, -0.02E-3] | **-0.06E-3** [-0.02E-2, -0.04E-4] |
| minP | **0.03E-1** [0.01E-1, 0.03E-1] | **-0.03** [-0.05, -0.01] |
| sT | **0.07E-6** [0.05E-6, 0.01E-5] | **0.01E-5** [0.05E-6, 0.02E-5] |
| sP | **0.09E-2** [-0.03E-3, 0.03E-1] | **-0.07E-1** [-0.02, -0.02E-1] |

**Figure S5. Density feedback and maximum climate variables for high-quality data subsets.**

Model probabilities (left panels, Table S6) and standardized *w*AIC*_c_*-averaged effect sizes (right panels, Table S7) after contrasting nine models with strength of compensatory density feedback from time series of abundance (response) and combinations of 6 explanatory variables including time-series length (*q*, years), temperature of the hottest month (maxT, °C), precipitation of the wettest month (maxP, mm), seasonality of temperature (sT = sd, °C) and seasonality of precipitation (sP = CV). Models were fitted as phylogenetic generalized least-squares regressions for two datasets comprising 91 bird and 55 mammal species, respectively.

**
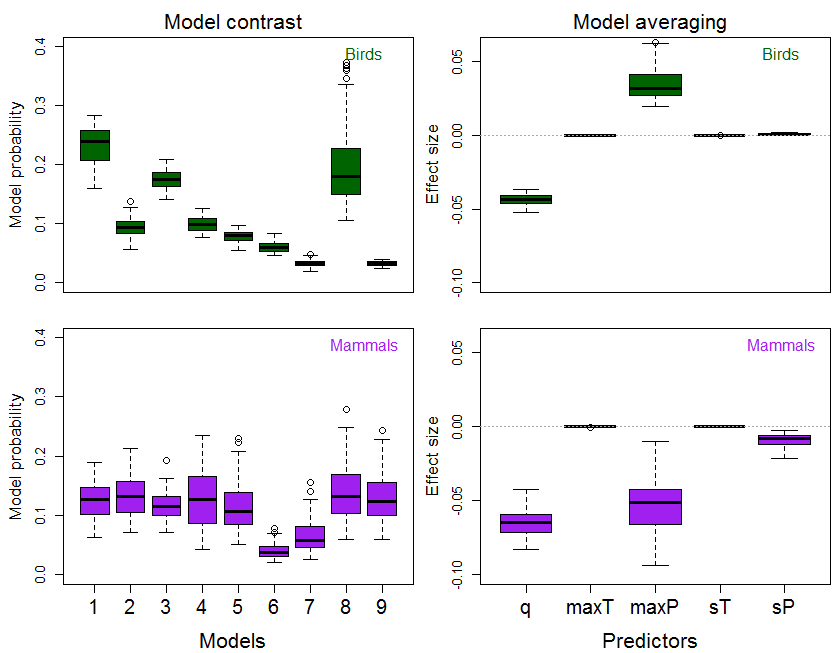
**

**Table S6. Density feedback and maximum climate variables for high-quality data subsets.**

Akaike’s information criterion (AIC*_c_*) support for the model set correlating temperature and precipitation variables^1^ to strength of compensatory density feedback for birds (76 species) and mammals (44 species) (Figure S5). All models were fitted using phylogenetic generalized least-squares regression, and model-ranking descriptors (*w*AIC*_c_*, %variance and *ER*)^2^ are medians from 100 bootstrapped samples [95^th^ percentile ranges].

| **taxa** | **top-ranked models** | ***w*AIC*_c_*** | **%variance** | ***ER*** | **top rank** |
| --- | --- | --- | --- | --- | --- |
| birds | Strength ~ *q* | **0.24** [0.17, 0.28] | **3.7** [3.1, 4.3] | - | **69** (25) |
|  | Strength ~ *q* + maxT | **0.09** [0.06, 0.12] | **3.7** [3.1, 4.3] | 2.6 | **0** (0) |
|  | Strength ~ *q* + maxP | **0.17** [0.14, 0.20] | **3.7** [3.0, 4.2] | 1.4 | **2** (51) |
|  | Strength ~ *q* + sT | **0.10** [0.08, 0.12] | **3.6** [3.0, 4.2] | 2.4 | **0** (0) |
|  | Strength ~ *q* + sP | **0.08** [0.06, 0.09] | **3.7** [3.1, 4.3] | 3.0 | **0** (0) |
|  | Strength ~ *q* + maxT + maxP | **0.06** [0.04, 0.08] | **3.7** [3.0, 4.2] | 4.0 | **0** (0) |
|  | Strength ~ *q* + maxT + sP | **0.03** [0.02, 0.04] | **3.7** [3.0, 4.3] | 7.2 | **0** (0) |
|  | Strength ~ *q* + sT + maxP | **0.18** [0.11, 0.36] | **3.4** [2.9, 4.0] | 1.4 | **29** (2) |
|  | Strength ~ *q* + sT + sP | **0.03** [0.03, 0.04] | **3.6** [3.0, 4.2] | 7.4 | **0** (0) |
|  |  |  |  |  |  |
| mammals | Strength ~ *q* | **0.13** [0.07, 0.19] | **2.1** [1.8, 2.4] | 1.0 | **12** (13) |
|  | Strength ~ *q* + maxT | **0.13** [0.08, 0.20] | **2.0** [1.7, 2.3] | - | **25** (14) |
|  | Strength ~ *q* + maxP | **0.12** [0.08, 0.16] | **2.0** [1.7, 2.3] | 1.1 | **1** (11) |
|  | Strength ~ *q* + sT | **0.13** [0.06, 0.23] | **2.0** [1.6, 2.3] | 1.1 | **12** (29) |
|  | Strength ~ *q* + sP | **0.11** [0.06, 0.21] | **2.0** [1.7, 2.2] | 1.2 | **18** (5) |
|  | Strength ~ *q* + maxT + maxP | **0.04** [0.02, 0.07] | **2.0** [1.7, 2.3] | 3.4 | **0** (0) |
|  | Strength ~ *q* + maxT + sP | **0.06** [0.03, 0.12] | **1.9** [1.6, 2.2] | 2.4 | **0** (0) |
|  | Strength ~ *q* + sT + maxP | **0.13** [0.07, 0.24] | **1.8** [1.5, 2.1] | 1.1 | **17** (15) |
|  | Strength ~ *q* + sT + sP | **0.12** [0.06, 0.22] | **1.8** [1.5, 2.0] | 1.0 | **15** (13) |

**^1^ maxT** = temperature of the hottest month (°C), **maxP =** precipitation of the wettest month (mm); **sT =** seasonality of temperature (sd, °C), and **sP =** seasonality of precipitation (CV). The model set equated *q* as control variable (i.e., present in all models), eight combinations of climate variables [maxT | maxP | sT | sP | maxT+maxP | maxT+sP | sT+maxP | sT+sP], and a null model with only *q*.

**^2^ *w*AIC*_c_*** = model probabilities given each dataset and model set; %**variance** = % variance in density-feedback strength explained by each model within the set; ***ER*** = evidence ratio of first model over the remaining models according to *w*AIC*_c_*; and **top rank** = times each model was top-ranked over the 100 bootstrapped samples (times each model was second-ranked).

**Table S7. Density feedback and maximum climate variables for high-quality data subsets.**

Standardized model-averaged effect sizes of time-series length (*q*, years), mean temperature of the hottest month (maxT, °C), mean precipitation of the wettest month (maxP, mm), seasonality of temperature (sT = sd, °C) and seasonality of precipitation (sP = CV) as explanatory variables of variation in strength of compensatory density feedback (response) in birds (77 species) or mammals (45 species). Statistical models were fitted as phylogenetic generalized least-squares regressions, with a total of nine models in each contrasted set (Figure S5, Table S6). Effect sizes are medians (in bold) for 100 bootstrapped samples [95^th^ bootstrapped percentile ranges].

| **variable** | **birds** | **mammals** |
| --- | --- | --- |
| *q* | **-0.03** [-0.04, -0.02] | **-0.05** [-0.08, -0.01] |
| maxT | **0.01E-5** [-0.06E-4, -0.05E-4] | **-0.6E-3** [-0.02E-2, -0.04E-4] |
| maxP | **0.08** [0.05, 0.12] | **-0.03** [-0.05, -0.01] |
| sT | **0.03E-5** [0.02E-5, 0.04E-5] | **0.01E-5** [0.05E-6, 0.02E-5] |
| sP | **0.03E-2** [-0.03E-3, 0.08E-2] | **-0.07E-1** [-0.02, -0.02E-1] |

**Generalized linear (with/without mixed-effects) models, including (Text S2) properties of the dataset, modelling approach and results**

**Text S2. Dataset Properties, Modelling Approach and Results for GLM/GLMM Analyses.**

**Dataset Properties**

The dataset included climate variables and time series of abundance of 160 populations (98 birds, 62 mammals) from 149 species (92 birds, 57 mammals) from 108 different localities in 28 countries, while 86 % of the populations were from the Northern Hemisphere (mainly Europe and North America). This dataset was slightly larger than that used in the PGLS. The additional time series of abundance included three species that were not present in the global phylogenies of birds and mammals [[8](#_ENREF_8),[9](#_ENREF_9)] , and 11 additional populations (i.e., populations of the same species from different localities) of species already included in our PGLS. The median time-series length was 31 years with 95^th^ percentile ranges of 10 to 93. We extracted climate variables as for the PGLS analyses, and replicated all analyses for the total sample of 160 populations, and a high-quality subset of 132 populations (82 birds, 50 mammals; Figure S1B) from 124 species (74 birds, 50 mammals), 94 localities and 27 countries, after meeting the high-quality criteria for data selection following Herrando-Pérez et al. [[3](#_ENREF_3)] (see above).

**Modelling Approach**

We used the same model set fitted through PGLS, except that two control variables were included in all models: time-series length and body size (Table S8). We collected maximum body length recorded in the wild for each species from experts and online sources (e.g., www.bto.org, www.demogr.mpg.de/longevityrecords, genomics.senescence.info/species). We used body length for consistency with previous research [[3](#_ENREF_3)]. Body length and body mass were strongly correlated in our dataset, and we did fit all models using body mass, with identical results (results not shown). Finally, alternatively to the PGLS approach, we also controlled for phylogenetic non-independence by allowing different intercepts for species grouped by higher taxonomic levels [[10](#_ENREF_10)]. We did so by including the Linnaean taxonomic level of *order* as a random factor in each model of our set (controls by *class* in the model set gave identical model support and similar fixed effects); lower taxonomic ranks were not possible to model due to many *families* and *genuses* represented by only one species); while taxonomic classification followed the IUCN template (www.iucnredlist.org). We fitted our data using generalized linear mixed-effects models (GLMM) for the full dataset [[11](#_ENREF_11)] and generalized linear models (GLM) for birds and mammals separately. We met the assumptions of normality and homoscedasticity (checked in QQ and residual plots) with the response being on a proportional scale through a square-root-transformation, which was supported by a likelihood-based test of Box & Cox [[12](#_ENREF_12)]. We replicated the analyses for all taxa, the subsets of mammals and birds separately, and the high-quality data subsets of all taxa, mammals and birds. Low sample sizes for specific geographical areas or taxonomical orders precluded further data partitions. Model ranking, bootstrapping and standardized, model-averaged fixed effects were calculated as in the PGLS. Bootstrapping was based on the 26 localities that contributed more than one population/species (20 % of the dataset) ⎯ see main text for details.

**Table S8. Model set used for GLMM and GLM analyses.**

All models include the same response, i.e., strength of compensatory density feedback across taxa (fitted by GLMM and OR = taxonomic *order* as random factor), and bird and mammal species (fitted by GLM, no phylogenetic random effect). Control variables were included in all models, namely *q* = length of time series, and *body* = body size. Climate variables encompassed: mT = annual temperature (mean, in °C), mP = annual precipitation (mean, in mm), sT = seasonality of temperature (standard deviation, in °C), and sP = seasonality of precipitation (coefficient of variation).

| Model 1: strength ~ *q* + *body* + (1 \| OR) |
| --- |
| Model 2: strength ~ *q* + *body* + mT (1 \| OR) |
| Model 3: strength ~ *q* + *body* + mP (1 \| OR) |
| Model 4: strength ~ *q* + *body* + sT (1 \| OR) |
| Model 5: strength ~ *q* + *body* + sP (1 \| OR) |
| Model 6: strength ~ *q* + *body* + mT + mP (1 \| OR) |
| Model 7: strength ~ *q* + *body* + mT + sP (1 \| OR) |
| Model 8: strength ~ *q* + *body* + sT + mP (1 \| OR) |
| Model 9: strength ~ *q* + *body* + sT + sP (1 \| OR) |

**Results**

The null model (including only the control variables of time-series length and body size) was top-ranked in all model contrasts (Figures S6, S7), and explained 34.8 (high-quality data subset) to 36.1 % (all taxa) of the deviance in density-feedback strength across populations (all taxa) and 38,7 to 42.5 % and 44,1 to 47.4 % for birds and mammals, respectively (Tables S9, S11). This null model was top-ranked in 82 to 86 % (all taxa), 86 to 89 % (birds) and 44 to 74 % (mammals) of the bootstrapped samples, and had 19 to 20 (all taxa), 10 to 12 (birds) and 2 to 8 (mammals) times more median information-theoretic support than any second-ranked model including climate vaiables (Tables S9, S11), based on the evidence ratio of the respective AIC*_c_* probabilities (*w*AIC*_c_*). The next-best-ranked model included the fixed effect of mean annual precipitation (mP), wherein mP explained 1.0 % (full dataset) to 1.8 % (high-quality subset) of the deviance in strength for all taxa and 3.7 % to 4.3 % for birds (Figures S6, S7, Tables S9, S11). For mammals, the best non-control explanatory variable was the seasonality of precipitation (sP) for the full dataset and mean temperature (mT) for the high-quality subset, explaining 5.5 % to 10.4 % of the deviance, respectively (Figures S6, S7, Tables S9, S11).

In support of the model rankings above, median model-averaged effect sizes (standardized over all explanatory variables) were all close to zero for the four environmental variables (Figures S6, S7, Tables S10, S12). The effect size of body size varied from -0.04 (birds) to -0.03 (all taxa and mammals) for the full dataset (Figure S6; Table S10), and from -0.03 (all taxa and birds) to -0.02 (mammals) for the high-quality subset (Figure S7, Table S12), so density-feedback strength increased from large to small body sizes across species.

We replicated all analysis with the model sets including extreme climate variables (as in the PGLS): (*i*) maximum precipitation of the wettest and maximum temperature of the hottest month, and (*ii*) minimum precipitation of the driest and minimum temperature of the coldest month. Our results (not shown) were upheld, and the majority of variation in density-feedback strength was explained by the control variables, mostly body size.

**Figure S6. Density feedback, mean climate variables and model contrasts using linear models.**

Model probabilities (left panels, Table S9) and standardized AIC*_c_*-averaged effect sizes (right panels, Table S10) after contrasting 9 models (Table S8) with strength of compensatory density feedback from time series of abundance (response) and combinations of 6 explanatory variables including time-series length (*q*, years), body size (*body*, mm), mean annual temperature (mT, °C), mean annual precipitation (mP, mm), seasonality of temperature (sT = sd, °C) and seasonality of precipitation (sP = CV). Statistical models were fitted as generalized linear mixed-effects models (random effect = Linnean taxonomical *order*) for all taxa (160 populations: 98 birds + 62 mammals) and as generalized linear models for the subsets of birds (92 species) and mammals (57 species).

**Table S9. Density feedback, mean climate variables and model contrasts using linear models.**

Akaike’s information criterion (AIC*_c_*) support for the first- and second-ranked models correlating temperature and precipitation variables^1^ to strength of compensatory density feedback for all taxa, and only mammals or birds (160 populations, 152 species). All models were fitted under a generalized linear mixed-effects framework for all taxa and included three control variables, namely time-series length (*q*, years), body size (*body*, mm) and the taxonomic level of *order* as random factor. We used generalized linear models (i.e., without a random factor) for the data subsets of birds and mammals separately. Model-ranking descriptors (*w*AIC*_c_*, %DE and *ER*)^2^ are medians from 100 bootstrapped samples [95^th^ percentile ranges].

| **dataset** | **top-ranked models** | ***w*AIC*_c_*** | **%DE** | ***ER*** | **top Rank** |
| --- | --- | --- | --- | --- | --- |
| all taxa | Strength ~ *q* + *body* | **0.84** [0.17, 0.93] | **36.1** [15.1, 51.4] | 19 | **82** (18) |
|  | Strength ~ *q* + *body* + mP | **0.04** [0.01, 0.53] | **37.1** [16.9, 52.5] |  | **3** (40) |
| birds | Strength ~ *q* + *body* | **0.82** [0.04, 0.91] | **42.5** [16.4, 62.3] | 10 | **86** (12) |
|  | Strength ~ *q* + *body* + mP | **0.08** [0.02, 0.86] | **46.2** [22.1, 65.2] |  | **11** (67) |
| mammals | Strength ~ *q* + *body* | **0.38** [0.004, 0.82] | **47.4** [19.1, 62.6] | 2 | **44** (38) |
|  | Strength ~ *q* + *body* + sP | **0.21** [0.01, 0.98] | **56.5** [31.7, 74.7] |  | **31** (26) |

**^1^ mT** = mean annual temperature (°C), **mP =** mean annual precipitation (mm); other factors that did not emerge in top models were **sT =** seasonality of temperature (sd, °C), and **sP =** seasonality of precipitation (CV). The model set equated *q*, *body* and *order* (random factor) as control variables (i.e., present in all models), eight combinations of climate variables [mT | mP | sT | sP | mT+mP | mT+sP | sT+mP | sT+sP], and a null model with only *q* and *body* (Table S8).

**^2^ *w*AIC*_c_*** = model probabilities given each data and model sets; %**DE** = % deviance in density-feedback strength explained by each model within the set; ***ER*** = evidence ratio of first- over second-ranked model *w*AIC*_c_*; and **top rank** = times each model was top-ranked over the 100 bootstrapped samples (times each model was second-ranked).

**Table S10. Density feedback, mean climate variables and model contrasts using linear models.**

Standardized model-averaged effect sizes of time-series length (*q*, years), body size (*body*, mm), mean annual temperature (mT, °C), mean annual precipitation (mP, mm), seasonality of temperature (sT = sd, °C) and seasonality of precipitation (sP = CV) as explanatory variables of variation in strength of compensatory density feedback, for all taxa, and only mammals or birds (160 populations, 152 species). Statistical models were fitted as generalized linear mixed effects (all taxa) or generalized linear models (birds or mammals, separately), with a total of 9 models in each contrasted set (Table S8). Effect sizes are medians (in bold) for 100 bootstrapped samples [95^th^ bootstrapped percentile ranges].

| **variable** | **all taxa** | **birds** | **mammals** |
| --- | --- | --- | --- |
| *q* | **-0.05** [-0.08, -0.04E-1] | **-0.06** [-0.12, -0.03] | **-0.06E-2** [-0.07, 0.07] |
| *body* | **-0.03** [-0.04, -0.02] | **-0.04** [-0.08, -0.01] | **-0.03** [-0.05, -0.01] |
| mT | **-0.07E-7** [-0.05E-2, -0.04E-6] | **-0.05E-5** [-0.03E-3, 0.06E-4] | **-0.04E-3** [-0.06E-2, -0.09E-5] |
| mP | **0.06E-2** [-0.02E-1, 0.03] | **0.03E-1** [-0.05E-1, 0.01E-1] | **-0.01E-2** [-0.02, 0.02] |
| sT | **0.03E-9** [-0.02E-10, 0.02E-7] | **0.09E-9** [-0.04E-9, 0.04E-5] | **0.02E-9** [0.04E-11, 0.08E-8] |
| sP | **-0.06E-2** [-0.02, 0.02E-2] | **0.04E-2** [-0.02E-1, 0.07E-1] | **-0.01E-1** [-0.03, 0.02E-2] |

**Figure S7. Density feedback, mean climate variables and model contrasts using linear models for the high-quality data subsets.**

Model probabilities (left panels) and standardized AIC*_c_*-averaged effect sizes (right panels) after contrasting 9 models (Table S8) with strength of compensatory density feedback from time series of abundance (response) and combinations of 6 explanatory variables including time-series length (*q*, years), body size (body, mm), mean annual temperature (mT, °C), mean annual precipitation (mP, mm), seasonality of temperature (sT = sd, °C) and seasonality of precipitation (sP = CV). Statistical models were fitted as generalized linear-mixed effects models (random effect = Linnaean taxonomical *order*) for all taxa (132 populations: 82 birds + 50 mammals) and as generalized linear models for the subsets of birds (74 species) and mammals (50 species).

**Table S11. Density feedback, mean climate variables and model contrasts using linear models for the high-quality data subsets.**

Akaike’s information criterion (AIC*_c_*) support for the first- and second-ranked models correlating temperature and precipitation variables^1^ to strength of compensatory density feedback for all taxa, and only mammals or birds (132 populations, 124 species). All models were fitted under a generalized linear mixed effects framework for all taxa and included three control variables, namely time-series length (*q*, years), body size (*body*, mm) and the taxonomic level of *order* as random factor. We used generalized linear models (i.e., without a random factor) for the data subsets of birds and mammals separately. Model-ranking descriptors (*w*AIC*_c_*, %DE and *ER*)^2^ are medians from 100 bootstrapped samples [95^th^ percentile ranges].

| **dataset** | **top-ranked models** | ***w*AIC*_c_*** | **%DE** | ***ER*** | **top rank** |
| --- | --- | --- | --- | --- | --- |
| all taxa | strength ~ *q* + *body* | **0.84** [0.11, 0.93] | **34.8** [11.7, 53.4] | 20 | **86** (12) |
|  | strength ~ *q* + *body* + mP | **0.04** [0.005, 0.38] | **36.0** [15.3, 54.3] |  | **2** (45) |
| birds | strength ~ *q* + *body* | **0.83** [0.16, 0.92] | **38.7** [10.8, 60.8] | 12 | **89** (10) |
|  | strength ~ *q* + *body* + mP | **0.06** [0.02, 0.59] | **43.0** [12.6, 62.7] |  | **4** (59) |
| mammals | strength ~ *q* + *body* | **0.74** [0.06, 0.91] | **44.1** [20.1, 67.7] | 8 | **74** (20) |
|  | strength ~ *q* + *body* + mT | **0.08** [0.007, 0.77] | **54.5** [29.8, 75.8] |  | **21** (29) |

**^1^ mT** = mean annual temperature (°C), **mP =** mean annual precipitation (mm); other factors that did not emerge in top models were **sT =** seasonality of temperature (sd, °C), and **sP =** seasonality of precipitation (CV). The model set equated *q*, *body* and *order* (random factor) as control variables (i.e., present in all models), eight combinations of climate variables [mT | mP | sT | sP | mT+mP | mT+sP | sT+mP | sT+sP], and a null model with only *q* and *body*.

**^2^ *w*AIC*_c_*** = model probabilities given each data and model sets; %**DE** = % deviance in density-feedback strength explained by each model within the set; ***ER*** = evidence ratio of first- over second-ranked model *w*AIC*_c_*; and **top rank** = times each model was top-ranked over the 100 bootstrapped samples (times each model was second-ranked).

**Table S12. Density feedback, mean climate variables and model contrasts using linear models for the high-quality data subsets.**

Standardized model-averaged effect sizes of time-series length (*q*, years), body size (*body*, mm), mean annual temperature (mT, °C), mean annual precipitation (mP, mm), seasonality of temperature (sT = sd, °C) and seasonality of precipitation (sP = CV) as explanatory variables of variation in strength of compensatory density feedback (response), for all taxa, and only mammals or birds (132 populations, 124 species). Statistical models were fitted as generalized linear mixed effects (all taxa) or generalized linear models (birds or mammals, separately) (Table S8), with a total of nine models in each contrasted set (Table S8) Effect sizes are medians (in bold) for 100 bootstrapped samples [95^th^ bootstrapped percentile ranges].

| **Variable** | **all taxa** | **birds** | **mammals** |
| --- | --- | --- | --- |
| *q* | **-0.04** [-0.08, 0.02] | **-0.06** [-0.12, -0.0] | **-0.02E-2** [-0.11, 0.08] |
| *body* | **-0.03** [-0.04, -0.02] | **-0.03** [-0.07, -0.01E-1] | **-0.02** [-0.05, -0.01] |
| mT | **-0.06E-8** [-0.05E-6, -0.01E-9] | **-0.05E-5** [-0.03E-3, 0.01E-3] | **-0.06E-3** [-0.07E-2, -0.03E-4] |
| mP | **0.05E-2** [-0.02E-1, 0.03] | **0.02E-1** [-0.01E-1, 0.09] | **-0.05E-2** [-0.01, 0.01] |
| sT | **0.05E-12** [0.01E-13, 0.07E-10] | **0.01E-9** [0.09E-10, 0.05E-5] | **0.02E-9** [-0.14E-10, 0.07E-8] |
| sP | **-0.05E-2** [-0.01, 0.04E-2] | **0.02E-2** [-0.02E-1, 0.03] | **-0.01E-1** [-0.03, 0.04E-2] |

**References**

1. Kottek M, Grieser J, Beck C, Rudolf B, Rubel F (2006) World Map of the Köppen-Geiger climate classification updated. Meteorologische Zeitschrift 15: 259-263.

2. Beck C, Grieser J, Kottek M, Rubel F, Rudolf B (2005) Characterizing global climate change by means of Köppen climate classification. Klimastatusbericht: Deutscher Wetterdienst. pp. 139-149.

3. Herrando-Pérez S, Delean S, Brook BW, Bradshaw CJA (2012) Strength of density feedback in census data increases from slow to fast life histories. Ecology and Evolution 2: 1922-1934.

4. Turchin P, Taylor AD (1992) Complex dynamics in ecological time series. Ecology 73: 289-305.

5. Royama T (1992) Analytical population dynamics; Usher MB, Kitching RL, editors. London, UK: Chapman & Hall. 371 p.

6. Berryman AA (1999) Principles of population dynamics and their application. Cheltenham, UK: Stanley Thorners Ltd. 243 p.

7. Cook RD (1982) Residuals and influence in regression. New York, USA: Chapman & Hall.

8. Jetz W, Thomas GH, Joy JB, Hartmann K, Mooers AO (2012) The global diversity of birds in space and time. Nature 491: 444-448.

9. Bininda-Emonds ORP, Cardillo M, Jones KE, MacPhee RDE, Beck RMD, et al. (2007) The delayed rise of present-day mammals. Nature 446: 507-512.

10. Blackburn TM, Duncan RP (2001) Determinants of establishment success in introduced birds. Nature 414: 195-197.

11. Breslow NE, Clayton DG (1993) Approximate inference in generalized linear mixed models. Journal of the American Statistical Association 88: 9-25.

12. Box GEP, Cox DR (1964) An analysis of transformations (with discussion). Journal of the Royal Statistical Society B 26: 211-252.
